# Quantitative proteomic characterization and comparison of T helper 17 and induced regulatory T cells

**DOI:** 10.1371/journal.pbio.2004194

**Published:** 2018-05-31

**Authors:** Imran Mohammad, Kari Nousiainen, Santosh D. Bhosale, Inna Starskaia, Robert Moulder, Anne Rokka, Fang Cheng, Ponnuswamy Mohanasundaram, John E. Eriksson, David R. Goodlett, Harri Lähdesmäki, Zhi Chen

**Affiliations:** 1 Turku Centre for Biotechnology, University of Turku and Åbo Akademi University, Turku, Finland; 2 Turku Doctoral Programme of Molecular Medicine, University of Turku, Turku, Finland; 3 Department of Computer Science, Aalto University, Espoo, Finland; 4 Cell Biology, Biosciences, Faculty of Science and Engineering, Åbo Akademi University, Turku, Finland; 5 Department of Pharmaceutical Sciences, University of Maryland School of Pharmacy, Baltimore, Maryland, United States of America; University of Pennsylvania Perelman School of Medicine, United States of America

## Abstract

The transcriptional network and protein regulators that govern T helper 17 (Th17) cell differentiation have been studied extensively using advanced genomic approaches. For a better understanding of these biological processes, we have moved a step forward, from gene- to protein-level characterization of Th17 cells. Mass spectrometry–based label-free quantitative (LFQ) proteomics analysis were made of in vitro differentiated murine Th17 and induced regulatory T (iTreg) cells. More than 4,000 proteins, covering almost all subcellular compartments, were detected. Quantitative comparison of the protein expression profiles resulted in the identification of proteins specifically expressed in the Th17 and iTreg cells. Importantly, our combined analysis of proteome and gene expression data revealed protein expression changes that were not associated with changes at the transcriptional level. Our dataset provides a valuable resource, with new insights into the proteomic characteristics of Th17 and iTreg cells, which may prove useful in developing treatment of autoimmune diseases and developing tumor immunotherapy.

## Author summary

T helper 17 (Th17) cells and induced regulatory T (iTreg) cells are two subsets of T helper cells differentiated from naïve cells that play important roles in autoimmune diseases, immune homeostasis, and tumor immunity. The differentiation process is achieved by changes in numerous proteins, including transcription regulators, enzymes, membrane receptors, and cytokines, which are critical in lineage commitment. To profile protein expression changes in Th17 and iTreg cells, we polarized murine naïve CD4+ T (Thp) cells in vitro to Th17 and iTreg cells and performed quantitative proteomic analysis of these cells. More than 4,000 proteins, covering almost all subcellular compartments, were detected. Quantitative comparison of the protein expression profiles resulted in the identification of proteins specifically expressed in the Th17 and iTreg cells. Importantly, our combined analysis of proteome and gene expression data revealed protein expression changes that were not associated with changes at the transcriptional level. The present study serves as a valuable resource that may prove useful in developing treatment of autoimmune diseases and cancer.

## Introduction

Interleukin-17 secreting T helper (T helper 17 [Th17]) cells are involved in neutrophilia, tissue remodeling and repair, and production of antimicrobial proteins. In addition, they play a critical role in inflammation and autoimmunity. Regulatory T (Treg) cells are immunosuppressive and essential for maintaining self-tolerance and homeostasis. Natural regulatory T (nTreg) cells develop in the thymus and are therefore distinct from the cells undergoing parallel thymic differentiation to become the naïve progenitors of T helper 1 (Th1), T helper 2 (Th2), and Th17 cells [1–4]. However, naïve CD4+ T (Thp) cells can be induced in vitro to differentiate into cells with similar characteristics as Treg cells, which are defined as induced regulatory T (iTreg) cells [5–7]. In recent years, considerable interest has been directed toward the targeting of Th17 and/or Treg cells in the treatment of autoimmune diseases and developing tumor immunotherapy [8]. An understanding of the molecular mechanisms of Th17 and iTreg cell differentiation, together with identification of the key molecules in Th17 and iTreg cell function, will help to develop strategies to target or manipulate these two Th cell types.

Characterization of the molecular mechanisms directing the differentiation of naïve Th cells toward their distinct subsets—namely, Th1, Th2, Th17, and Treg cells—has been studied in some depth by using transcriptomic and epigenomic strategies [9–14]. However, the regulation of gene expression is also controlled at the posttranscriptional, translational, and posttranslational levels. Accordingly, poor levels of concordance between changes in protein abundance and mRNA expression have been reported, for example, with variation in mRNA quantities accounting for only around 40% of differences at the protein level [15, 16]. As proteins contribute the structural and functional elements of cells, a comprehensive view of the Th17/iTreg cells proteome is thus required. While protein expression profiling in Th1, Th2, and Treg cells has been reported [17–19] [20], there are no large-scale proteomic reports on Th17 cells. In the past, due to lower sensitivity and poorer reproducibility, cellular protein profiling was mostly limited to description of the higher and moderately abundant cellular components, and relatively less protein expression changes were quantified. Importantly, several of the signature molecules of Th cell subsets, such as key transcription factors (TFs) and cytokine receptors, are often present at low levels [21] and were difficult to detect with previous proteomics profiling approaches. However, with continued improvements in proteomic technology, the achievable levels of coverage have begun to approach those from genomic analyses, indicating detection of thousands of proteins from a single analysis. In particular, mass spectrometry coupled with liquid chromatography (LC-MS) provides an integrated system for analyzing protein components with improved sensitivity and moderate throughput.

In this study, label-free quantitative (LFQ) proteomics analysis was used to define the proteins from in vitro cultured murine Th17 and iTreg cells. Over 4,000 proteins were detected, with representation of almost all subcellular compartments. A shared protein signature was observed in the proteomes of the T cell receptor (TCR)-activated helper T cell (Th0), Th17, and iTreg cells, in addition to distinct differences in the proteins expressed in these two subsets. Importantly, protein expression changes were found that were not otherwise implicated from transcriptomics data. Pathway enrichment and network analysis improved the understanding of the functional context and organization of proteins in Th17 and iTreg cells. Our dataset provides a valuable resource for studying Th cell specification and further developing novel therapies to treat autoimmune diseases, inflammation, and tumors by manipulating or targeting Th17 or iTreg cells.

## Results

### Proteomic profiles of polarizing Th17 and iTreg cells

**Fig 1:**
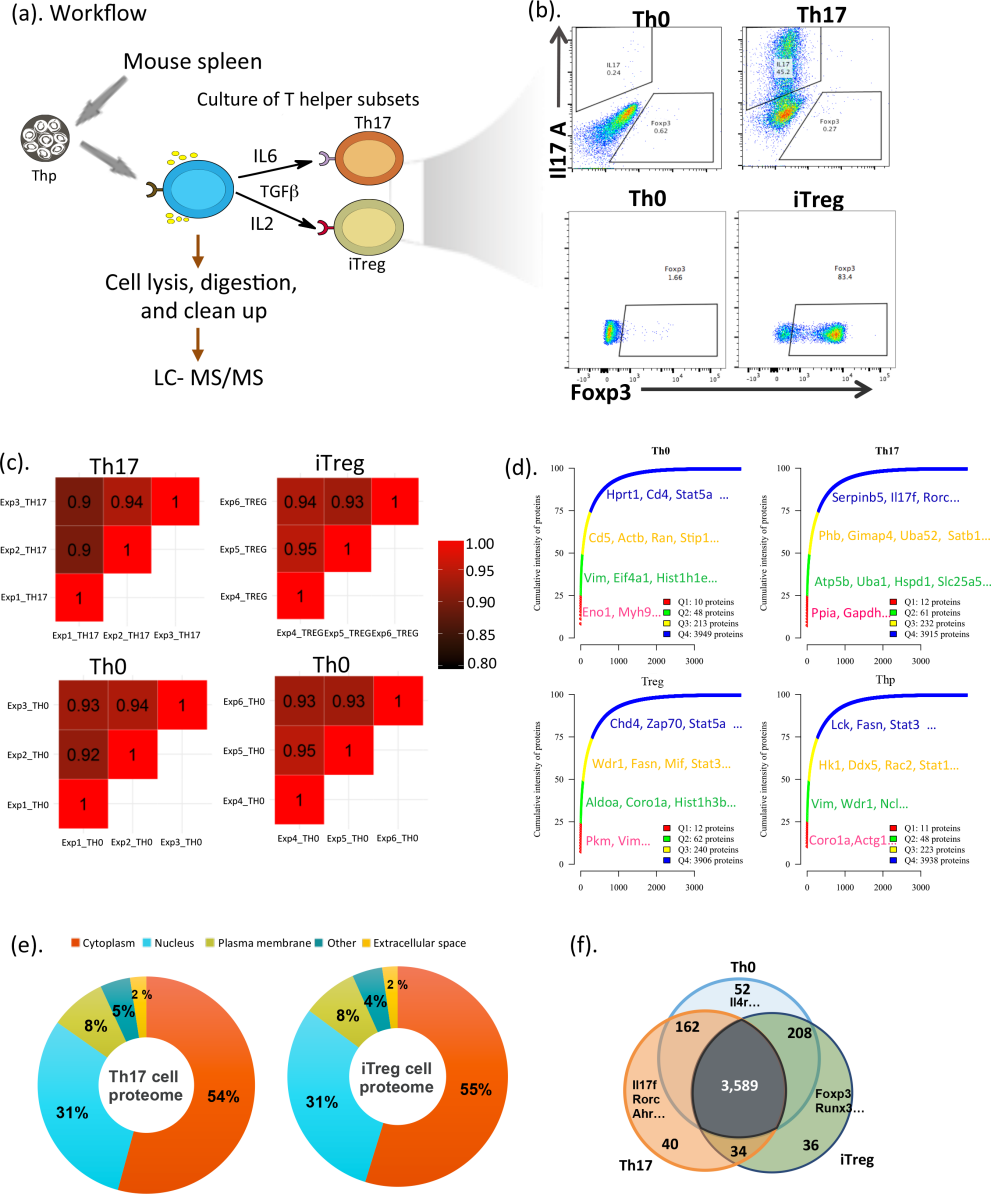
Th17 and iTreg cell proteome. (a) Illustration of experimental and proteomic workflow in the study. (b) Representative flow cytometry plots showing the expression of murine IL17 in Th0 and Th17 cells cultured for 3 d and Foxp3 in Th0 and iTreg cells cultured for 7 d followed by restimulation with anti-CD3/CD28 and polarizing cytokines for additional 3 d. Percentages of positive cells were indicated. (c) Pearson’s correlation plots showing the correlation value of biological triplicates for Th17, iTreg, Th0 paired with Th17, and Th0 paired with iTreg cells. (d) Cumulative protein abundances plotted against ranked proteins. The number of proteins in each quantile was shown on the lists and in S1 Data. (e) Pie chart with percentages of proteins identified across different cell compartments in Th17 and iTreg cells. The complete lists of proteins are in S1 Data. (f) Venn diagram with quantified proteins across Th0, Th17, and iTreg cells. The complete lists of proteins are in S1 Data. Actb, β-actin; Actg1, γ-actin 1; Ahr, aryl hydrocarbon receptor; Aldoa, aldolase A; Atp5b, ATP synthase subunit beta; Chd4, chromodomain helicase DNA-binding protein 4; Coro1a, coronin 1A; Ddx5, DEAD-box helicase 5; Eif4a1, eukaryotic translation initiation factor 4a1; Eno1, enolase 1; Fasn, fatty acid synthase; Foxp3, forkhead box P3; Gapdh, glyceraldehyde-3-phosphate dehydrogenase; Gimap4, GTPase IMAP family member 4; Hist1h1e; histone cluster 1 H1 family member E; Hk1, hexokinase 1; Hprt, hypoxanthine phosphoribosyltransferase; Hspd1, heat shock protein family D1; IL2, interleukin 2; Il4r, interleukin 4 receptor; IL6, interleukin 6; Il17 A, interleukin 17 A; IL17f, interleukin 17f; iTreg, induced regulatory T; Lck, lymphocyte cell-specific protein tyrosine kinase; LC-MS/MS, liquid chromatography–tandem mass spectrometry; Mif, macrophage migration inhibitory faction; Myh9, myosin heavy chain 9; Ncl, nucleolin; Phb, prohibitin; Pkm, pyruvate kinase M; Ppia, peptidylprolyl isomerase A; Rac2, ras-related C3 botulinum toxin substrate 2; Ran, Ras-related nuclear protein; Rorc, retinoic acid receptor–related orphan receptor C; Runx3, runt-related transcription factor 3; Satb1, special AT-rich sequence-binding protein 1; Serpinb5, Serpin Family B Member 5; Slc25a2, solute carrier family 25 member 2; Stat1, signal transducer and activator of transcription 1; Stat3, signal transducer and activator of transcription 3; Stat5a, signal transducer and activator of transcription 5A; Stip1, stress-induced phosphoprotein 1; TGFβ, transforming growth factor β; Th0, T cell receptor–activated helper T; Th17, T helper 17; Uba1, ubiquitin 1; Uba52, ubiquitin 52; Vim, vimentin; Wdr1, WD repeat domain 1; Zap70, zeta chain of T cell receptor–associated protein kinase 70.

To identify the proteome of Th17 cells, Thp cells were isolated and polarized toward Th17 cells for 72 h using standard culture protocols. Interleukin 2 (IL2) was not added to the in vitro Th17 cultures on account of its reported suppression of Th17 polarization [22]. Under these conditions, approximately 40% of interleukin 17 (IL17)-producing cells were established after 72 h of polarization. The 72 h time point was therefore chosen to study the proteome of polarizing Th17 cells. To compare the proteomics of the two closely linked Th cell subsets iTreg and Th17 cells, iTreg cells were polarized for 7 d followed by restimulation and polarization for another 3 d, after which 87% of the cells expressed forkhead box P3 (Foxp3) (Fig 1A and 1B). The expression of IL17 and Foxp3 were detected by intracellular protein staining followed by flow cytometry analysis of the Th17- and iTreg-polarized cells, respectively (Fig 1B). Following cell lysis with NP-40 and trypsin digestion, the samples were analyzed by liquid chromatography–tandem mass spectrometry (LC-MS/MS) (Fig 1A). Overall, from the analyses of 3 independent cultures of polarizing Th17, iTreg, activated (Th0) and Thp cells, 4,287 protein groups were detected. Principle component analysis showed that the Th17, iTreg, and Th0 subsets could be distinguished based on their protein expression profiles in all 3 of the independent cultures (S1A Fig). Furthermore, high Pearson’s correlation coefficients were observed for the 3 independent cultures (0.9–0.95) in all the cultured subsets (Fig 1C and S1B Fig). The identified proteins span a concentration range of 6 orders of magnitude, indicating detection of proteins across a wide range of expression (S1C Fig and S1 Data). To obtain an overview of the detected T helper cell proteomes, the cumulative contribution of each protein to total detected proteins was plotted. The 3 in vitro cultured Th cell subsets showed similar patterns for the protein cumulative intensities, in which the first quarter was mostly attributed to the cytoskeleton and glycolytic enzymes (Fig 1D and S1 Data). Analysis of protein location in terms of the cellular compartment indicated about half of the detected proteins were cytoplasmic and 31% were from the nucleus. Cytokines were identified amongst the 2% of proteins that were categorized as located from the extracellular space, such as interleukin 17F (IL17F) and interleukin 16 (IL16) in Th17 cells (Fig 1E and S1 Data). Cytokines and cytokine receptors are important for fate decision of Th cell differentiation. In order to facilitate the determination of plasma membrane proteins, the nonionic detergent NP-40 was used for sample preparation in this study, resulting in the identification of 317 and 312 plasma membrane proteins in Th17 or iTreg cells, respectively (Fig 1E and S1 Data). Importantly, some cytokine receptors and integrins—such as interleukin 2 receptor alpha (IL2RA), interleukin 2 receptor gamma (IL2RG), tumor necrosis factor receptor superfamily member 18 (TNFRSF18), intercellular adhesion molecule 1 (ICAM1), intercellular adhesion molecule 2 (ICAM2), integrin alpha L (ITGAL), integrin beta 1 (ITGB1), integrin beta 2 (ITGB2), and integrin beta 7 (ITGB7)—were detected both in Th17 and iTreg cells (S1 Data). As shown in Fig 1F and S1 Data, 3,589 proteins—including the expected CD3E, CD3G, CD2, CD4, CD5, CD6, CD28, CD44, and CD69—were detected in cells cultured under all of the 3 polarizing conditions. This supported the concept that although these cells are polarizing toward distinct functional subsets, they still carry a basic T cell signature. Furthermore, the analysis of these data revealed that proteins from almost all cellular compartments were detected, adding to the value of these data as a resource to further study candidate proteins potentially contributing to Th17 and iTreg cell differentiation and function.

### Protein expression changes during Th17/iTreg cell differentiation

**Fig 2:**
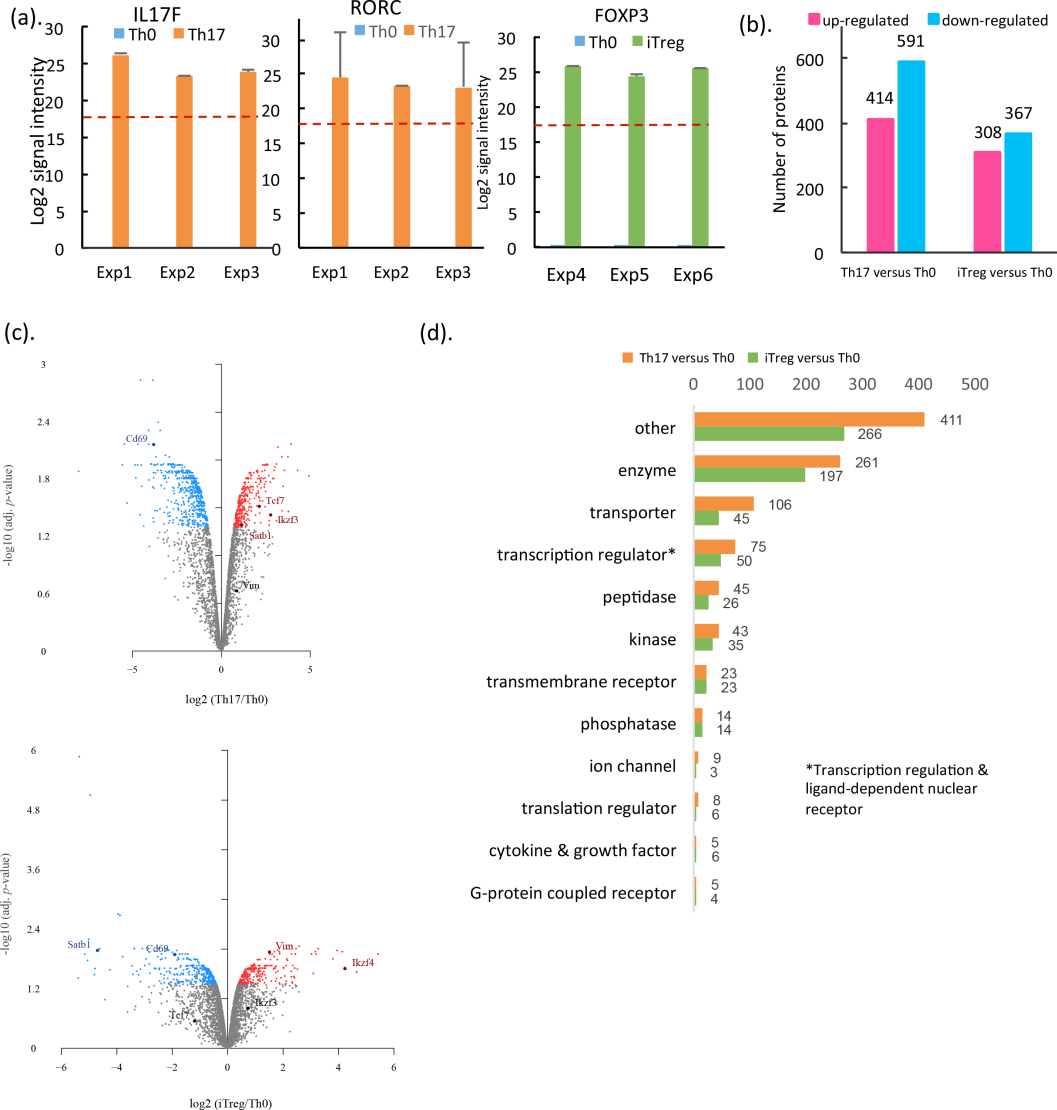
Protein Exp changes during Th17 and iTreg cell differentiation. (a) LC-MS/MS log2 median intensity values of cytokine IL17F, TF Rorc in Th0 and Th17, and TF Foxp3 in Th0 and iTreg cells from 3 replicates. Data shown are median values from 3 technical replicates (in S2 Data) with the SEM. Dotted line showing the minimum of LC-MS/MS signal intensity detections. (b) Number of DE proteins of Th17 and iTreg in comparison to Th0 cells. The complete lists of DE proteins can be found in S2 Data. (c) Volcano plots of statistical significance against fold-change of proteins between cell types (in S2 Data). Blue and red dots indicate statistically differentially abundant proteins. (d) Functional groups of significantly DE proteins in Th17 or iTreg cells compared to Th0 cells. Functional groups were annotated by using IPA (Qiagen Bioinformatics. www.qiagenbioinformatics.com). The complete lists of DE proteins are in S2 Data. DE, differentially expressed; Exp, expression; Foxp3, forkhead box P3; IKZF3, IKAROS family zinc finger 3; IKZF4, IKAROS family zinc finger 4; IL17F, interleukin 17F; IPA, Ingenuity Pathway Analysis; iTreg, induced regulatory T; LC-MS/MS, liquid chromatography–tandem mass spectrometry; Rorc, retinoic acid receptor–related orphan receptor C; Satb1, special AT-rich sequence-binding protein 1; SEM, standard error of the mean; Tcf7, transcription factor 7; TF, transcription factor; Th0, T cell receptor–activated helper T; Th17, T helper 17; Vim, vimentin.

Since Th17 and Treg cells play an important role in autoimmunity and inflammation [8, 23–25], detailed gene expression profiling studies have been carried out in them [10, 14, 26–28]. However, there are no reports using proteomics to profile protein expression during Th17 cell differentiation. In this study, quantitative proteomics of Th17, iTreg, and Th0 cells enabled the comparison of protein expression in differentiating Th17 and iTreg cells. Of the proteins detected in this study, 93% were detected in all 3 T cell subsets. However, 40 proteins were selectively expressed in response to Th17 polarizing cytokines (Fig 1F). Among these, cytokine IL17F and TF retinoic acid receptor–related orphan receptor C (RORC) have been previously well characterized as Th17 cell signature molecules [29–31]. Both of these were detected only in Th17 cells and not in activated Th0 cells (Fig 2A and S2 Data), confirming that our in vitro cultured Th cells were successfully polarized. The other proteins detected only in Th17 cells included aryl hydrocarbon receptor (AHR) and phosphodiesterase 5A (PDE5A). Amongst these, in the PDE5A gene, the active enhancer mark H3K27ac was detected in PMA-Ionomycin-stimulated Th17 primary cells [32]. Solute carrier family 4 member 2 (SLC4A2, also known as AE2) was also only detected in Th17 cells. It has been reported that the activity of SLC4A2 is sensitive to pH, and its mutation is associated with primary biliary cirrhosis and autoimmune disease of the urogenital tract [33, 34]. However, whether these genes play roles in Th17 cells has not been thoroughly studied. When comparing proteins detected in Th17 cells with TCR-activated Th0 cells, we found that there were statistically significant changes (false discovery rate [FDR] < 0.05) in the expression of 25.9% of all detected proteins in Th0 and Th17 cells (1,005 out of 3,880 proteins) (see Methods). Amongst these, the expression of 414 proteins—including IL17F, RORC, AHR, and IKAROS family zinc finger 3 (IKZF3)—were up-regulated, whereas 591 proteins—such as TNFRSF18 (GITR) and tumor necrosis factor receptor superfamily member 4 (TNFRSF4, OX40)—were down-regulated (Fig 2B and 2C upper panel, S2 Data).

The role of iTreg cells is to control the effector cells (including Th17, Th1, or Th2 cells) and to suppress immune response [2, 8]. Foxp3 is the key TF for iTreg cell subset differentiation, as well as having a suppressive function [8, 23]. With the proteomics analysis of in vitro cultured iTreg cells, FOXP3 protein was detected in all 3 independent cultures, whereas it was not in TCR-activated Th0 cells (Fig 2A and S2 Data). Comparing the proteomes of Th0 cells and polarized iTreg cells, in addition to FOXP3, the expression of, for example, runt-related transcription factor 3 (RUNX3), integrin subunit alpha E (ITGAE), G protein unit gamma 2 (GNG2), and vimentin (VIM) were also up-regulated, whereas the expression of such proteins as special AT-rich sequence-binding protein 1 (SATB1) and CD69 were down-regulated (Fig 2C upper panel, S2 Data). Altogether, quantitative proteomics from 3 independent in vitro cell cultures revealed expression changes of 675 proteins after 10 d differentiation toward iTreg cells compared to Th0 cells (FDR < 0.05). On the basis of functional annotation with Ingenuity Pathway Analysis (IPA, QIAGEN Bioinformatics, https://www.qiagenbioinformatics.com), the differentially expressed (DE) proteins were classified into 12 functional groups (Fig 2D and S2 Data). Similarly as with Th17 cells, the enzyme was the functional group represented with the most protein expression changes when compared to Th0 cells.

Following the activation via TCR and costimulatory receptors, the fate decision of Th cell subsets is largely determined by TFs. After 72 h of in vitro polarization of Th17 cells and 10 d of polarization of iTreg cells, the expression of 75 and 50 transcription regulators and ligand-dependent nuclear receptors were significantly changed, respectively (Fig 2D, S2A Fig and S2 Data), including RORC, FOXP3, AHR, nuclear factor of activated T cells 2 (NFATC2), and IKZF3. Some of these differentially expressed TFs, such as CCR4-NOT transcription complex subunit 2 (CNOT2) and family with sequence similarity 129 member B (FAM129B), were proteins whose expression level was induced after both differentiation of Th17 and iTreg cells. As transcription regulators, their functions in Th17/iTreg cell differentiation are largely unknown. Further functional characterization of these TFs or identification of their target genes in Th17/iTreg cells may help to understand transcriptional mechanisms of the Th17/iTreg cell differentiation process. Kinases are important contributors to cell fate decisions. Many protein kinase inhibitors have been developed to treat diseases such as cancer, inflammation, and autoimmune disorders [35, 36]. In our data, both in Th17 and iTreg cells, we found the protein expression of a group of kinases was changed, such as mitogen-activated protein kinase 11 (MAPK11), right open reading frame kinase 1 (RIOK1), and cleavage and polyadenylation factor I subunit 1 (CLP1) (Fig 2D, S2 Data). Many of these have not been previously reported in the context of Th17/iTreg cell function or differentiation. Follow-up studies on these kinases or candidate proteins from the other functional groups (Fig 2D, S2 Data) could provide further insight on the development of novel therapeutic targets to modify Th17/Treg-mediated diseases.

Pathway enrichment analysis of the DE proteins detected from the comparison of polarizing Th17 and Th0 cells indicated that their association with a number of different biological processes. Notably, oxidative phosphorylation (OXPHO), antigen processing and presentation and metabolic pathways were the top enriched pathways (S2B Fig; S2 Data), suggesting that the Th17 cell differentiation process is accompanied with antigen processing and metabolic changes, and especially OXPHO. Pathway analysis in the DE proteins from the comparison of iTreg and Th0 cells only revealed 2 enriched pathways, systemic lupus erythematosus and alcoholism, which both included changes of several histone cluster 1 h2 and h4 family members (S2 Data).

### Distinct protein expression changes in iTreg and Th17 cells

**Fig 3:**
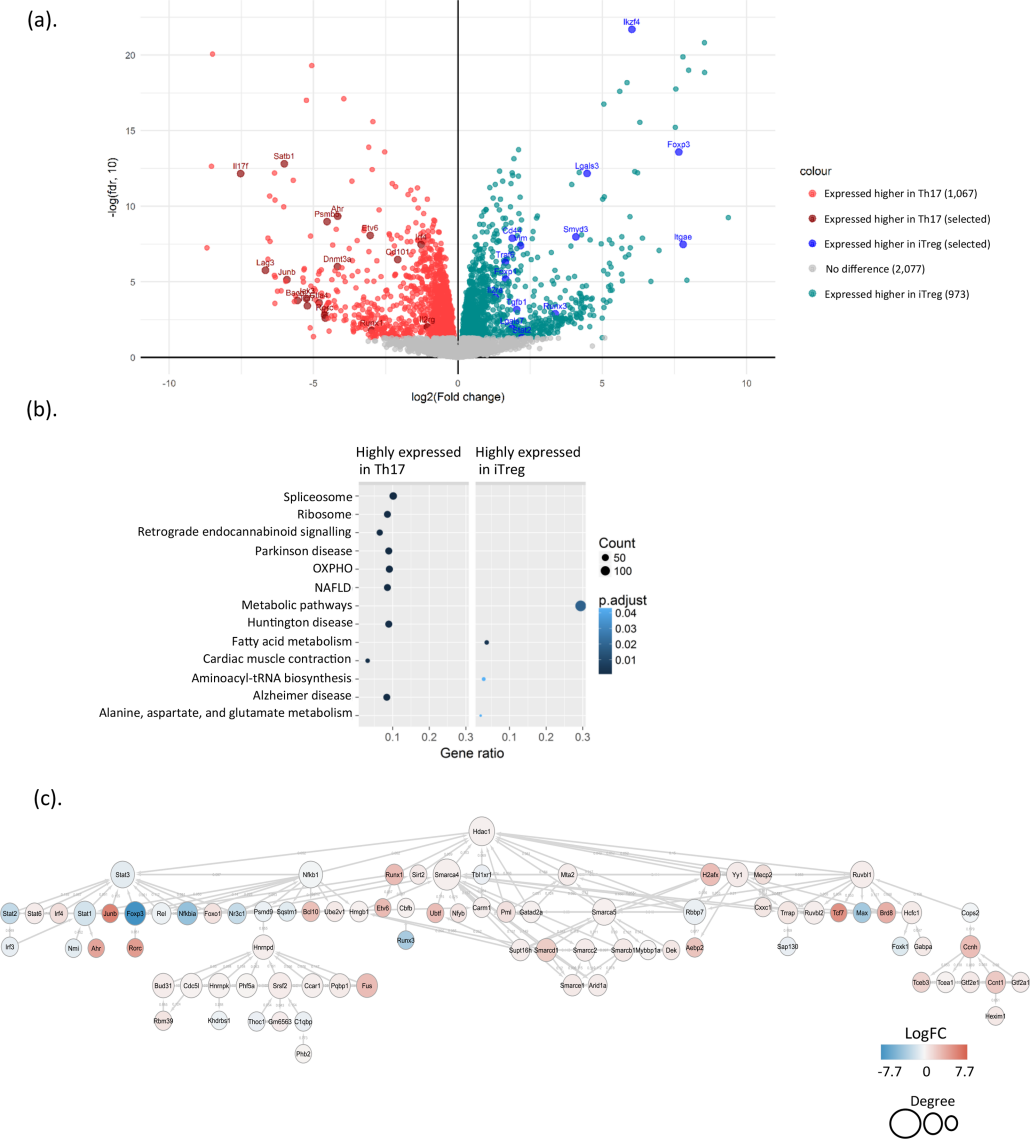
DE proteins in Th17 and iTreg cell differentiation. (a) Volcano plot of the comparison of proteome from Th17 versus iTreg cell. Green and red dots indicate statistically DE proteins. The complete lists of DE proteins in Th17 and iTreg cells are in S3 Data. (b) KEGG pathway analysis was performed on up-regulated (highly expressed in Th17) and down-regulated (highly expressed in iTreg) proteins in Th17 versus iTreg comparison. The pathways presented in the plot are significantly (Benjamini-Hochberg adjusted *p* < 0.05) enriched; size and color of dot indicate the number of proteins detected for that pathway and adjusted *p*-value respectively. The lists of pathways and proteins are in S3 Data. (c) Transcriptional regulatory network of Th17 and iTreg cells is shown. The TF annotation for DE proteins in comparison of Th17 and iTreg cells was obtained using IPA. The lists of TFs are in S3 Data. Hierarchical layout of String network is displayed. The red and blue nodes indicate proteins highly expressed in Th17 and iTreg cells, respectively. The size of nodes indicates the degree of connectivity of the nodes. Aebp2, adipocyte enhancer–binding protein 2; Ahr, aryl hydrocarbon receptor; Arid1a, AT-rich interaction domain 1A; Bach, BTB domain and CNC homology; Bcl10, B cell chronic lymphocytic leukemia/lymphoma 10; Brd8, bromodomain-containing 8; C1qbp, complement C1q binding protein; Carm1, coactivator-associated arginine methyltransferase 1; Cbfb, core binding factor beta; Ccar1, cell cycle and apoptosis regulator 1; Ccnh, cyclin H; Ccnt1, cyclin T1; Cdc5l, cell division cycle 5–like protein; Cops2, constitutive photomorphogenesis 9 signalosome subunit 2; Ctla4, cytotoxic T lymphocyte–associated protein 4; Cxxc1, CXXC finger protein 1; DE, differentially expressed; Dnmt3a, DNA methyltransferase 3 alpha; Etv6, ETS Variant 6; fdr, false discovery rate; Foxk1, forkhead box K1; Foxo1, forkhead box O1; Foxp1, forkhead box P1; Foxp3, forkhead box P3; Fus, Fus RNA binding protein; Gabpa, GA-binding protein transcription factor subunit alpha; Gatad2a, GATA zinc finger domain containing 2A; Gtf2a1, general transcription factor IIA subunit 1; Gtf2e1, general transcription factor IIE subunit 1; H2afx, histone 2A family member X; Hcfc1, host cell factor C1; Hdac1, histone deacetylase 1; Hexim1, hexamethylene bisacetamide inducible 1; Hmgb1, high-mobility group box 1; Hnrnpd, heterogeneous nuclear ribonucleoprotein D; Hnrnpk, heterogeneous nuclear ribonucleoprotein K; IKZF4, IKAROS family zinc finger 4; Il2, interleukin 2; Il2ra, interleukin 2 receptor alpha; Il2rg, interleukin 2 receptor gamma; Il17f, interleukin 17f; IPA, Ingenuity Pathway Analysis; Irf3, interferon regulatory factor 3; Irf4, interferon regulatory factor 4; ITGAE, integrin subunit alpha E; iTreg, induced regulatory T; Jak3, Janus kinase 3; KEGG, Kyoto Encyclopedia of Genes and Genomes; Khdrbs1, KH RNA binding domain containing, signal transduction–associated 1; Lag3, lymphocyte activating 3; Lgals3, galectin 3; Lgals7, galectin 7; Max, MYC-associated factor X; Mecp2, methyl-CpG binding protein 2; Mta2, metastasis-associated 1 family member 2; Mybbp1a, MYB binding protein 1A; Nfkbia, nuclear factor of kappa light polypeptide gene enhancer in B cells inhibitor alpha; Nfyb, nuclear transcription factor Y beta; Nmi, N-myc and STAT interactor; NR3C1, nuclear receptor subfamily 3 group C member 1; OXPHO, oxidative phosphorylation; Phb1, prohibitin 1; Phb2, prohibitin 2; Phf5a, PHD finger protein 5a; Pml, promyelocytic leukemia; Pqpb1, polyglutamine-binding protein 1; Psmb5, proteasome subunit beta 5; Psmd9, proteasome 26S subunit non-ATPase 9; Rbbp7, retinoblastoma binding protein 7; Rbm39, RNA binding motif 39; Rorc, retinoic acid receptor–related orphan receptor C; Runx1, runt-related transcription factor 1; Runx3, runt-related transcription factor 3; Ruvbl1, RuvB-like AAA ATPase 1; Ruvbl2, RuvB-like AAA ATPase 2; Sap130, Sin3A-associated protein 130; Satb1, special AT-rich sequence-binding protein 1; Sqstm1, sequestosome 1; Sirt2, sirtuin 2; Smarca4, SWI/SNF-related matrix-associated actin-dependent regulator of chromatin subfamily A member 4; Smarca5, SWI/SNF-related matrix-associated actin-dependent regulator of chromatin subfamily A member 5; Smarcb1, SWI/SNF-related matrix-associated actin-dependent regulator of chromatin subfamily B member 1; Smarcc2, SWI/SNF-related matrix-associated actin-dependent regulator of chromatin subfamily C member 2; Smarcd, SWI/SNF-related matrix-associated actin-dependent regulator of chromatin subfamily D; Smarce1, SWI/SNF-related matrix-associated actin-dependent regulator of chromatin subfamily E member 1; Smyd3, SET And MYND domain containing 3; Srsf2, serine- and arginine-rich splicing factor 2; Stat1, signal transducer and activator of transcription 1; Stat2, signal transducer and activator of transcription 2; Stat3, signal transducer and activator of transcription 3; Stat6, signal tranducer and activator of transcription 6; Supt16h, SPT16 homolog; Tbl1xr1, transducin beta–like 1 X-linked receptor 1; Tcea1, transcription elongation factor A polypeptide 1; Tceb3, transcription elongation factor B polypeptide 3; Tcf7, transcription factor 7; TF, transcription factor; Tgfbr-I, transforming growth factor beta receptor type 1; Th17, T helper 17; Thoc1, THO complex 1; Traf6, tumor necrosis factor receptor–associated factor 6; Trrap, transformation/transcription domain–associated protein; Ube2v1, ubiquitin conjugating enzyme E2 V1; Ubtf, upstream binding transcription factor; Vim, vimentin; Yy1, yin yang 1.

Although Th17 and iTreg cells have different functions, they share transforming growth factor β (TGFβ) as a common cytokine, which is required for both iTreg and Th17 cell differentiation. To gain further insight into these differences, we next evaluated which proteins showed distinct changes in these 2 cell lineages. Comparing the proteomes detected for the Th17 and iTreg cells, we found 2,040 proteins with a statistically significant (FDR < 0.05) expression difference. Of the 2,040 DE proteins, 1,067 proteins were expressed higher in Th17 cells, and 973 proteins were expressed higher in iTreg cells (Fig 3A and S3 Data). Similarly to FOXP3, which was highly up-regulated in iTreg cells, other TFs—such as IKAROS family zinc finger 4 (IKZF4) and RUNX3—were also highly expressed in iTreg cells. Importantly, we found that the chromatin organizer SATB1 was highly expressed in Th17 cells when compared to TCR-activated Th0 cells, and its expression changed in an opposite direction in cells cultured under Th17/iTreg polarizing conditions (Fig 3A and S3A and S3B Fig). The down-regulation of SATB1 in iTreg cells was consistent with a previous finding that expression of SATB1 was suppressed by Foxp3 [37], whilst in Th17 differentiating cells, we observed that it was up-regulated. Most interestingly, expression of another 19 proteins, such as ETS Variant 6 (ETV6) and transglutaminase 2 (TGM2), showed the same pattern as SATB1. In contrast, 26 proteins—including C-C motif chemokine receptor 7 (CCR7), brain abundant membrane attached signal protein 1 (BASP1), adenylosuccinate synthetase isozyme 1 (ADSSL1), enolase 3 (ENO3), RIOK1, fascin actin-bundling protein 1 (FSCN1), and myosin heavy chain 11 (MYH11)—were expressed in an opposite manner: repressed in Th17 and induced in iTreg cells (S3C Fig). It would be interesting to investigate whether and how these proteins contribute to Th17 and iTreg cell function.

Since Th17 cells produce inflammatory cytokines and since iTreg cells are immunosuppressive, in order to explore how the observed protein expression changes in Th17 and in iTreg cells are linked with the cell functions, we performed a Kyoto Encyclopedia of Genes and Genomes (KEGG) pathway enrichment analysis of DE proteins in the comparison of Th17 and iTreg cells. We found that several pathways were enriched in the proteins highly expressed in Th17 cells, including spliceosome, ribosome, and OXPHO (Fig 3B and S3 Data). Importantly, the most significantly enriched pathways for the proteins highly expressed in iTreg cells were the metabolic pathway and fatty acid metabolism pathways (Fig 3B and S3 Data). To further illustrate functional impact of protein expression differences in Th17 and iTreg cells, we constructed an enrichment map using core enrichment genes of the Gene Set Enrichment Analysis (GSEA) (S3D Fig). The protein–protein interactions were derived from the String database [38] using confidence level 0.7. As shown in S3D Fig, the module named “pathways whose core enrichment genes overlap with Oxidative phosphorylation” including a group of proteins such as ATP synthase subunit beta (ATP5B), ATP synthase F1 subunit alpha (ATP5A1), cyclooxygenase 2 (COX2), cyclooxygenase 5A (COX5A), and NADH:ubiquinone oxidoreductase core subunit S1 (NDUFS1) were highly expressed in Th17 cells and were closely associated with each other. Based on previous studies, these proteins have been involved in OXPHO as well as in Huntington disease, Alzheimer disease, Parkinson disease, retrograde endocannabinoid signaling, and nonalcoholic fatty liver disease (NAFLD) (Fig 3B, S3D Fig and S3 Data), whereas a group of proteins including acetyl-CoA acyltransferase 2 (ACAA2), aldehyde dehydrogenase 2 (ALDH2), hydroxyacyl-CoA dehydrogenase (HADH), carnitine palmitoyltransferase 2 (CPT2), and 3-hydroxyacyl-CoA dehydratase 3 (HACD3) were highly expressed in iTreg cells and were clustered as a module named fatty acid metabolism. This module comprised pathways of fatty acid metabolism, fatty acid elongation, and fatty acid degradation (S3D Fig, S3 Data). Additionally, the group of proteins involved in ribosome biogenesis was highly expressed in Th17 cells and formed the “ribosome” module. Results from pathway analysis indicate that Th17 and iTreg cells use a distinct energy resource to maintain their functions.

Among the DE proteins in the comparison of Th17 and iTreg cells, there were a group of 155 TFs and ligand-dependent nuclear receptors, including the 2 lineage-specific TFs, Foxp3 and RORC. In order to investigate the highly complex interaction patterns of these TFs in regulating Th17 and iTreg lineage commitment, we constructed a transcriptional regulatory network in Th17 and iTreg cells using these DE TFs. The histone deacetylase 1 (HDAC1) was responsible for the deacetylation of lysine residues on the N-terminal of the core histones (H2A, H2B, H3, and H4). HDAC1 was slightly but significantly more highly expressed in Th17 cells compared to iTreg cells (S3 Data). A study in human Th17 cells has indicated that HDAC1 interacts with and deacetylates retinoic acid receptor–related orphan receptor γ T (RORγt) [39]. Protein–protein association network analysis using String *Mus musculus* database showed the association of HDAC1 with multiple TFs, including signal transducer and activator of transcription 3 (STAT3), nuclear factor kappa B subunit 1 (NFKB1), runt-related transcription factor 1 (RUNX1), and sirtuin 2 (SIRT2) (Fig 3C). Given the essential role of histone deacetylation for epigenetic repression of gene expression, HDAC1 was at the highest regulatory level in the hierarchical view of the transcriptional regulatory network in Th17 and iTreg cells. The critical role of STAT3 in Th17 and iTreg differentiation has been established by several studies [22, 30, 40–43]. Consistent with previous findings, the network analysis demonstrated the association of STAT3 with other STATs as well as with interferon regulatory factor 4 (IRF4), JUNB, FOXP3, REL, forkhead box O1 (FOXO1), and nuclear receptor subfamily 3 group C member 1 (NR3C1) (Fig 3C). In the constructed network, the size of each node correlates with its connectivity. Interestingly, one of the biggest nodes in the network was SWI/SNF-related matrix-associated actin-dependent regulator of chromatin subfamily A member 4 (SMARCA4), which was expressed more highly in Th17 compared to iTreg cells (Fig 3C). It has been reported that Smarca4 was among the Th17 positive module. Knockdown of Smarca4 regulator down-regulated Th17 marker genes in Th17 cells [28]. Notably, in the constructed network, in addition to SMARCA4, several other members of the SWI/SNF family—including SWI/SNF-related matrix-associated actin-dependent regulator of chromatin subfamily A member 5 (SMARCA5), SWI/SNF-related matrix-associated actin-dependent regulator of chromatin subfamily D member 1 (SMARCD1), SWI/SNF-related matrix-associated actin-dependent regulator of chromatin subfamily B member 1 (SMARCB1), SWI/SNF-related matrix-associated actin-dependent regulator of chromatin subfamily C member 2 (SMARCC2), SMARCE1—were all highly expressed in Th17 cells (Fig 3C), suggesting the SWI/SNF family may play an import role in regulating Th17 differentiation or function. As such, the transcriptional regulatory network analysis not only confirmed previous findings but also predicts potential regulators that have not been previously appreciated.

Collectively, Th17 and iTreg cells have distinct protein expression profiles that are associated with differently enriched pathways. Pathway enrichment and network analysis improved the understanding of the functional context and organization of proteins in Th17 and iTreg cells. Follow up functional characterization of these molecules may provide novel potential targets influencing Th17/iTreg balance.

### Correlation of protein and RNA expression changes during Th17 and iTreg cell differentiation

**Fig 4:**
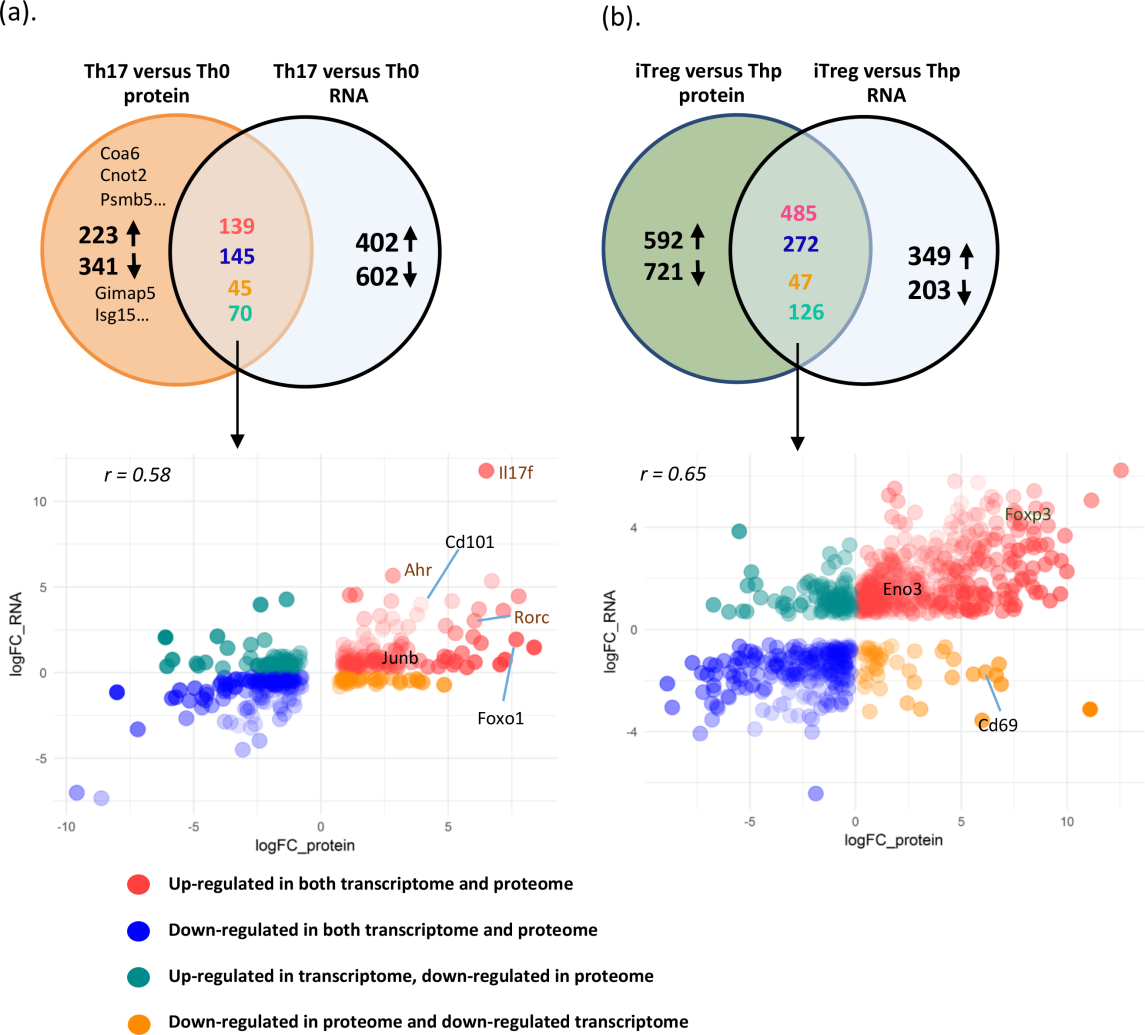
Correlation of protein and RNA expression changes during Th17 and iTreg cell differentiation. Venn diagram showing the comparison of DE proteins with corresponding transcripts and DE transcripts with encoded detected proteins in comparison of Th17 and Th0 cells (a) or iTreg versus Thp cells (b). Scatterplot of proteins that were observed in proteomic and transcriptomic comparison of Th17 versus Th0 cell (a) or iTreg versus Thp cells (b). The lists of detected proteins and transcripts in Th17 versus Th0 cells and in iTreg versus Thp cells are in S4 Data. Ahr, aryl hydrocarbon receptor; Cnot2, CCR4-NOT transcription complex subunit 2; Coa6, cytochrome c oxidase assembly factor 6; DE, differentially expressed; Eno3, enolase 3; Foxo1, forkhead box O1; Foxp3, forkhead box P3; Gimap5, GTPase IMAP family member 5; Il17f, interleukin 17F; Isg15, interferon-stimulated gene 15; iTreg, induced regulatory T; Psmb5, proteasome subunit beta 5; Rorc, retinoic acid receptor–related orphan receptor C; Th0, T cell receptor–activated helper T; Th17, T helper 17; Thp, naïve CD4+ T.

Characterization of molecular mechanisms involved in regulating Th17 cell differentiation has been studied at the transcriptional and epigenetic level [10, 26–28]. To identify protein expression changes not previously detected in mRNA expression–profiling studies, we generated an RNA-seq data set from in vitro cultured murine Th0 and Th17 cells with the same culture conditions as the proteomics study. Firstly, transcripts from 96.7% of the proteins detected by LC-MS/MS could be found in RNA-seq data (S4 Data). As expected, increased expression of IL17F and RORC in Th17 cells was observed both in quantitative proteomics as well as RNA-seq data. To address the question of which protein expression changes were not seen at mRNA level, we compared the DE genes and DE proteins (Th17 versus Th0). First, to make the proteomics and transcriptomics data comparable, from DE proteins we removed proteins without corresponding transcripts; similarly, the DE genes without detected corresponding proteins were also removed from DE genes. Interestingly, among the 963 DE proteins with corresponding detected transcripts in Th17 cells, expression changes for 284 (29.5%) proteins agreed with their gene expression changes detected by RNA-seq at the same time point (Fig 4A and S4 Data). This group included 139 molecules that were up-regulated consistently both at mRNA and protein level, such as Il17f, Rorc, and Ahr, and 145 molecules were down-regulated consistently both at mRNA and protein level, including, for example, Tnfrsf18, Cd44, Il2rg, and interferon-inducible GTPase 1 (Iigp1). Therefore, for these 284 genes, our proteomics experiments confirmed their mRNA expression changes at the protein level. Notably, among the 963 DE proteins with corresponding detectable transcripts in Th17 cells, 564 proteins (58.6%) were only found to be DE at the protein but not at mRNA level (Fig 4A, S4 Data) at the same time point. This is comparable with previously reported proteomics studies showing the correlation between mRNA and protein changes [44–47]. Based on protein annotation, the DE proteins with and without consistent mRNA expression changes showed similar composition of functional groups (S4A Fig and S4 Data). To address whether the DE genes encoding non-DE proteins were at relatively low expression levels, we checked the distribution of fragments per kilobase of transcript per million mapped reads (FPKM) values for the group of DE genes encoding DE proteins and the group of only DE genes encoding non-DE protein. We did not observe any major differences, indicating that DE genes encoding non-DE proteins were not relatively lowly expressed (S4B Fig and S4 Data). This indicates translation, protein degradation and export process, and maybe posttranslation modification play an important role in controlling Th17/iTreg cell protein expression. These newly identified DE proteins may also contribute to Th17 cell polarization and function. Given the importance of Th17 cells in immune response and disease pathogenesis, it is worth further characterizing their function in Th17 cells.

To compare proteomics and transcriptomics data from in vitro cultured iTreg cells with Thp cells, we used published microarray data [10] and observed increased Foxp3 expression in iTreg cells both at protein level as well as at mRNA level. Comparing our proteomics data with these microarray data generated from iTreg cells with the similar conditions, we observed 757 genes with consistent expression changes at protein and mRNA level. Among the overlapping DE proteins and DE mRNAs, the Pearson’s correlation coefficient was 0.65 (Fig 4B, S4 Data). However, we found that for 67.8% of the DE proteins, the changes were inconsistent with the RNA expression changes at the selected time point. Among the DE proteins in iTreg cells, 1,313 proteins were found to be DE at the protein but not the RNA level (Fig 4B, S4 Data)—such as stathmin 2 (STMN2), prolyl 4-hydroxylase subunit alpha 1 (P4HA1), and reactive oxygen species modulator 1 (ROMO1)—for which their contributions to iTreg cells have not yet been characterized. As another example, one of the H3K4 histone methyltransferases, SET and MYND domain containing 3 (SMYD3), was up-regulated only at the protein level. Notably, a recent study has reported that TGFβ induces SMYD3 expression during iTreg differentiation [48]. SMYD3 regulates Foxp3 expression by enhancing the trimethylated state of H3K4 in the promoter and the conserved noncoding DNA sequence 1 (CNS-1) element of Foxp3.

### Validation of DE proteins in Th17 and iTreg cells

**Fig 5:**
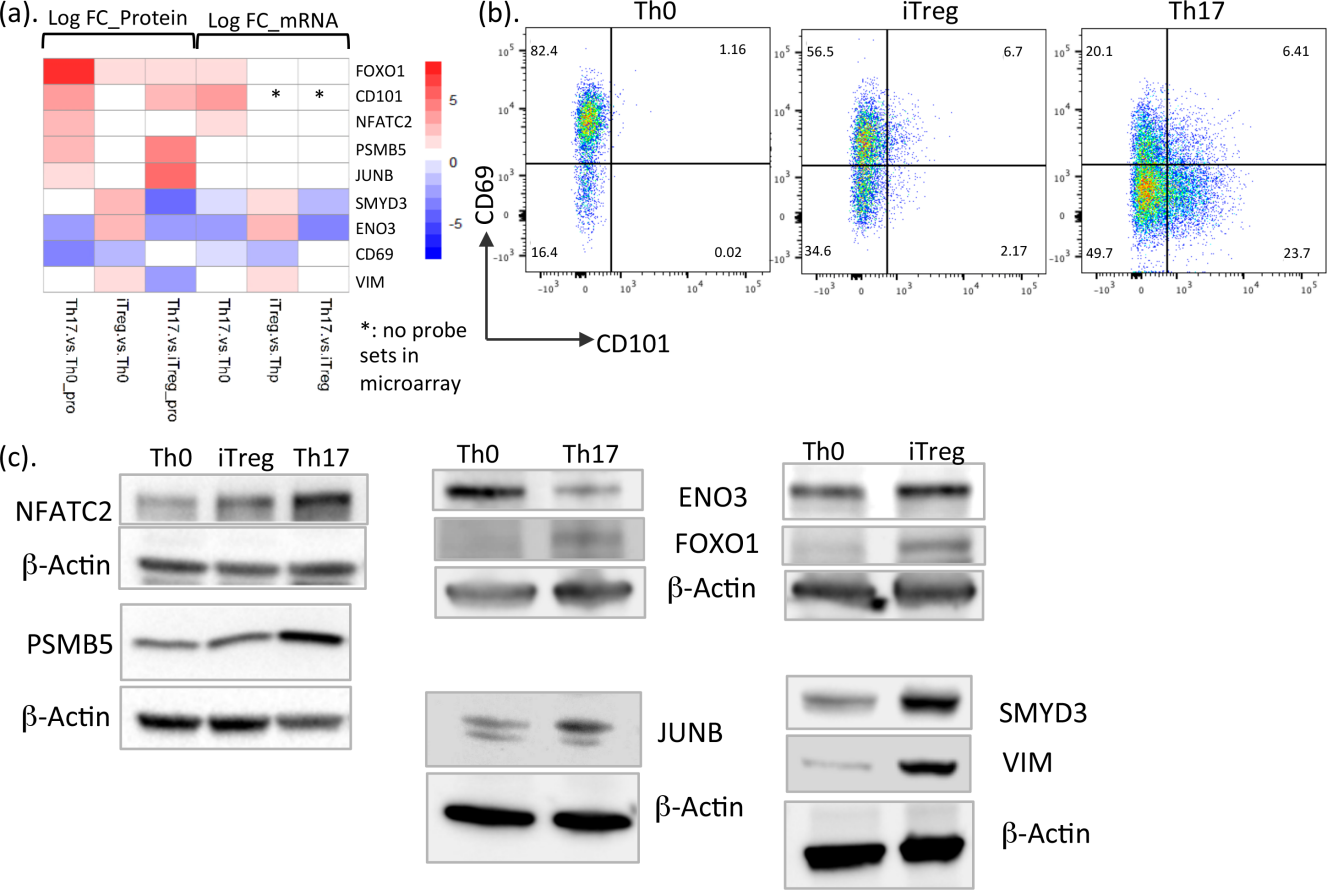
Validation of protein expression changes with different technologies. (a) Heatmap showing the log fold change values (in S5 Data) of proteins and mRNA DE in Th17 and iTreg cells in comparison with Th0 cells and Th17 compared with iTreg cells. (b) Flow cytometry analysis demonstrating the expression of surface molecules CD69 and CD101 in murine Th0, iTreg, and Th17 cells. One replicate is shown. (c) Immunoblot analysis of DE proteins in iTreg and Th17 cells compared to Th0 cells. Representative blots from 2–3 independent experiments are shown. DE, differentially expressed; ENO3, enolase 3; FOXO1, forkhead box O1; iTreg, induced regulatory T; NFATC2, nuclear factor of activated T cells 2; PSMB5, proteasome subunit beta 5; SMYD3, SET and MYND domain containing 3; Th0, T cell receptor–activated helper T; Th17, T helper 17; Thp, naïve CD4+ T; VIM, vimentin.

To confirm the LFQ results, we used either flow cytometry or western blot to quantify expression of selected proteins in 3 additional cultures. Comparing to TCR-activated Th0 cells in our primary proteomics data, we found expression of CD69 to be significantly down-regulated in both Th17 and iTreg cells (Fig 5A and S5 Data). CD69 is widely accepted as a marker of early T cell activation with an immune regulatory role [3, 4, 49, 50]. Compared to Th0 cells, after 72 h culture, CD69 expression was clearly down-regulated in iTreg and especially in Th17 cells (Fig 5B). CD101 is highly expressed in lymphoid and myeloid cells in intestinal tissues, and its expression in T cells was sufficient for Treg function and the inhibition of T cell proliferation [51, 52]. Notably, reduced expression of CD101 has been detected in inflammatory bowel disease (IBD) patients [52]. We found that Th17 culturing conditions induced CD101 expression and that it was expressed at higher levels in Th17 cells than iTreg cells (Figs 3A and 5A). This difference was also confirmed by flow cytometry (Fig 5B).

It has been previously shown that TF NFATC2 (NFAT1) interacts with FOXP3, and the complex suppresses IL2 production and Treg cell function [53]. We found that expression of NFATC2 was induced in Th17 cells from the transcriptomics as well as proteomics data (Fig 5A and S5 Data). Our western blot analyses also validated this expression change of NFATC2 in Th17 cells compared to Th0 cells (Fig 5C, S5 Fig).

We were especially interested in the proteins selectively expressed in iTreg or Th17 cells that may contribute to their lineage-specific functions. Expression levels of SMYD3 and ENO3 were up-regulated in iTreg cells (Fig 5A and S5 Data). Consistent with this observation, western blot analysis on 3 additional murine T cell cultures showed enhanced SMYD3 expression in iTreg cell (Fig 5C, S5 Fig). Protein expression of FOXO1 was increased in both iTreg cells and Th17 cells (Fig 5C, S5 Fig). Taken together, using different technologies, we validated selected protein expression changes detected by LFQ proteomics analysis, indicating the identified protein expression changes in this study are reliable, hence supporting the utility of this dataset to identify novel molecules putatively involved in the regulation of iTreg or Th17 cell function.

### VIM is highly expressed in iTreg cells and influences TGFβ-induced Foxp3 expression

**Fig 6:**
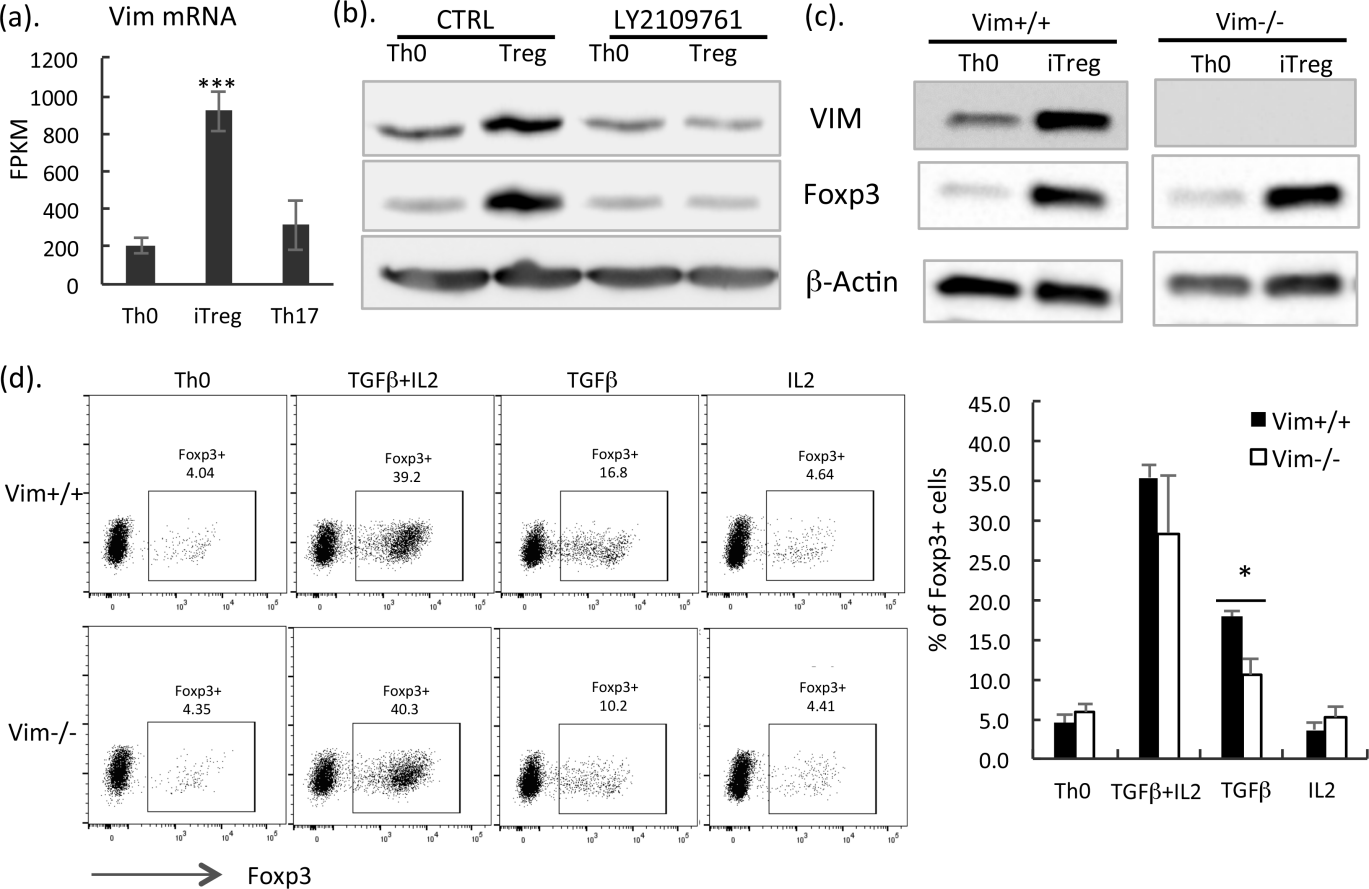
VIM is highly expressed in iTreg cells and influences TGFβ-induced Foxp3 expression. (a) mRNA expression of Vim from RNA-seq data generated in the present study. WT Thp cells were cultured under Th0, iTreg, and Th17 polarizing condition for 3 d. RNAs were isolated and processed for RNA-seq. Data shown are median FPKM values from 3 independent experiments (in S6 Data) with the SEM. Statistical analysis was performed by using paired Student *t* test. ***: *p* < 0.01. (b) Immunoblot analysis of Thp cells cultured to Th0 and iTreg with and without 1 μM LY2109761 for 3 d. VIM, Foxp3, and loading control **β**-actin were shown. Representative of 3 independent experiments is shown. (c) Thp cells cultured to Th0 and iTreg for 3 d. Vim, Foxp3, and loading control **β**-actin were shown. Representative blots of 3 independent experiments are shown. (d) Flow cytometry analysis of WT and Vim-deficient CD4+ T cells cultured with TCR activation (Th0) and with cytokines (IL2+ TGFβ1, TGFβ1, IL2) for 3 d. Representative intracellular cytokine staining for Foxp3 was shown on left panel, and percentage of Foxp3 expression cells detected from 4 independent experiments (in S6 Data) was shown on right panel. CTRL, control; Foxp3, forkhead box P3; FPKM, fragments per kilobase of transcript per million mapped reads; IL2, interleukin 2; iTreg, induced regulatory T; SEM, standard error of the mean; TCR, T cell receptor; TGFβ, transforming growth factor beta; Th0, T cell receptor–activated helper T cell; Th17, T helper 17; Thp, naïve CD4+ T; VIM, vimentin; WT, wild-type.

From both proteomics and transcriptomics data, we found that the intermediate filament protein VIM is highly expressed in iTreg (Fig 5C and S6 Data). In iTreg proteomics study, we cultured T cells for 10 d, to evaluate whether the VIM is up-regulated in early polarizing iTreg cells, and we extracted FPKM values of Vim mRNA expression from our RNA-seq data in which Thp cells were cultured under Th0, iTreg, and Th17 for 3 d. As shown in Fig 6A and S6 Data, expression of Vim was significantly up-regulated in polarizing iTreg cells compared to Th0 cells. Because TGFβ is the key cytokine to induce Foxp3 expression and iTreg differentiation, we tested whether the enhanced expression of VIM in iTreg cells was dependent on TGFβ. LY2109761 is a transforming growth factor β receptor type 1 (TGFβR-I) kinase inhibitor [54–56]. Consistent with the proteomics data, in western blot, we also detected high VIM expression in iTreg cells compared with Th0 (Fig 6B). Importantly, we observed that addition of LY2109761 diminished Foxp3 as well as VIM expression (Fig 6B), indicating that the up-regulation of VIM in iTreg cells is induced by TGFβ.

Next, we assessed whether VIM plays a role in regulating Foxp3 expression. Thp cells from wild-type (WT) and Vim−/− mice [57] were isolated and activated with plate-bound anti-CD3/CD28 and cultured under Th0 (anti-CD3/CD28) and iTreg (anti-CD3/CD28 combined with TGFβ+IL2) polarizing condition. We observed the absence of VIM in Vim−/− T cells and again detected enhanced expression of VIM in WT iTreg cells (Fig 6C). However, we did not observe significant change of Foxp3 expression in iTreg cells cultured from Vim−/− CD4+ T cells compared to control T cells (Fig 6C). To dissect the effect of VIM on TGFβ- and IL2-induced Foxp3 expression, we cultured control and Vim−/− Thp cells in the presence of only TGFβ, only IL2, or TGFβ combined with IL2 for 3 d. Results from intracellular Foxp3 staining showed significantly reduced Foxp3 expression in Vim−/− T cells cultured with TGFβ compared to control T cells (Fig 6D). Interestingly, after addition of IL2 and TGFβ or only with IL2 from 4 independent cultures in Vim−/− cells, no significant expression change of Foxp3 was detected (Fig 6D), indicating that IL2 prevented the effect of VIM in TGFβ-induced Foxp3 expression. Collectively, these data suggest that TGFβ up-regulates VIM expression in iTreg cells. VIM contributes to TGFβ-induced Foxp3 expression.

## Discussion

In recent years, next-generation sequencing technology has been widely applied to study the transcriptome of many cellular types and changes, including the differentiation of Th subsets [14, 27, 58, 59]. In the present study, the application of LFQ proteomics technology provided successful detection and LFQ of the master TF of Treg cells, Foxp3 in cultured iTreg cells, and the hallmark cytokine IL17F and TF RORC in Th17 polarizing cells. To our knowledge, this is the first quantitative proteome profile study in Th17 cells. Given the importance of Th17 and Treg cells in inflammation, autoimmunity, and cancer, development of novel strategies to modulate Th17/Treg cell balance to treat immune-associated diseases and cancer is a subject of interest.

In this study, we chose to study protein profiles of the Th17 cell after in vitro polarization for 3 d. As mentioned above, because of the suppressive effect of IL2 for Th17 differentiation [22], IL2 was not added to the Th17 polarizing cytokine cocktail. Under these conditions, using intracellular cytokine staining, IL17 was detected in approximately 40% of the polarizing Th17 cells. To achieve full polarization of the iTreg cells, we cultured Thp cells for 7 d and restimulated with anti-CD3/CD28 and cultured with TGFβ combined with IL2 for another 3 d. From 3 independent cultures, approximately 87% of cells express Foxp3. These conditions were subsequently chosen for the proteomics profiling of the Th17 and iTreg cells on account of the levels of polarization achieved.

For Th cell subsets, a large amount of gene expression data has been generated using microarray or more recently developed RNA-seq technology. Since intracellular protein levels are balanced through protein biosynthesis, degradation, and export, transcriptomics analysis cannot capture protein-level changes in these processes, and accordingly, many of the detected gene expression changes cannot be confirmed at protein level. Therefore, we took a step beyond mRNA measurement and used LFQ proteomics to construct a protein landscape for Th17 and iTreg cells. With the comparison of proteomics and transcriptomics data from cells cultured in vitro with the same conditions, we quantitatively confirmed 284 and 757 of the DE genes at the protein level in differentiated Th17 compared to Th0 and in iTreg compared to Thp cell, respectively. Some of these proteins have been identified in previous studies to play an important role in regulating Th17/iTreg cell differentiation, such as RORC, IL17F, Foxp3, CD101, NFATC2, mucosa-associated lymphoid tissue 1 (MALT1), and AHR. We also identified a panel of proteins—such as RNA exonuclease 2 (REXO2), paxillin (PXN), and FAM129B—with both RNA and protein expression changes in Th17 cells, whose role in Th17 cell differentiation or function yet needs to be defined. These proteins with consistent changes both at the RNA and protein level in Th17 and iTreg cells provide novel candidates for further functional characterization. Therefore, combining mRNA and protein profiling data can identify potentially relevant targeting molecules.

From further comparisons of the proteomics and transcriptomics data within the same cell types, we found that more than half of the protein-level changes were not detected at the mRNA level. For example, CNOT2 is a subunit of the CCR4-NOT complex, which regulates mRNA synthesis and degradation and is also involved in mRNA splicing, transport, and localization. CNOT2 interacts with histone deacetylases and functions as a repressor of polymerase II transcription [60]. It interacts with several subunits of the silencing mediator for retinoid and thyroid receptors (SMRT)/nuclear receptor corepressor (NCoR)-histone deacetylase 3 (HDAC3) complex and contributes to transcription repression [61]. However, as a transcription regulator, the function of CNOT2 in primary T cells, especially in the differentiation of Th subsets, is largely unknown. In the present study, we found for the first time that the CNOT2 protein is up-regulated in polarizing Th17 cells, but the similar induction of Cnot2 mRNA was not detected by RNA-seq at the same time point. This warrants further characterization of the function of CNOT2 in Th17 cell differentiation and its contribution to autoimmune inflammation.

In this study, pathway enrichment analysis of the proteins DE in Th17 and iTreg cells showed that the OXPHO pathway was enriched in proteins highly expressed in Th17 cells, whereas fatty acid metabolism pathway was enriched in proteins highly expressed in iTreg cells. In the past few years, several studies have demonstrated that the cellular metabolism pathways have a critical role in regulating Th17/iTreg cell differentiation [62–64]. It has been reported that hypoxia-inducible factor 1 subunit α (HIF1α) is up-regulated in Th17 cells and promotes glycolysis in Th17 differentiation [65]. The transport of glucose across the plasma membranes of mammalian cells is facilitated by the Glut family (also called solute carrier family 2). Studies have shown that effector T cells express higher level of glucose transporter 1 (GLUT1; solute carrier family 2 member 1 [SLC2A1]) than iTreg cells. On the other hand, iTreg cells utilize lipid oxidation as a primary metabolic pathway [63]. Moreover, it has been shown that Th17 cell proliferation and cytokine production are inhibited when glycolysis is blocked [65]. In our data, the glycolysis pathway was not enriched in proteins highly expressed in Th17 cells. However, we found that expression of another GLUT family member, glucose transporter 3 (GLUT3; solute carrier family 2 member 3 [SLC2A3]), but not GLUT1, was significantly higher in Th17 cells compared to iTreg cells. Further characterization of contribution of these 2 glucose transporters in Th17/iTreg balance is needed. OXPHO is an important metabolic process for generating ATP molecules in mitochondria. With the comparison of the Th17 and iTreg cell proteomics data, we detected a group of 52 proteins in the OXPHO pathway—including several COX molecules and NADH dehydrogenases—that were highly expressed. These molecules have also been found to be highly expressed in several neuron diseases, such as Parkinson disease, Huntington disease, and Alzheimer disease. To our knowledge, this is the first study reporting a big group of OXPHO-regulated protein changes in Th17 cells. Further functional characterization of this pathway in Th17 cells and the link with diseases seems to be important. Lastly, we found the enrichment of fatty acid metabolic pathway highly expressed in iTreg cells. In addition to the already reported carnitine palmitoyltransferase 1A (CPT1A) [18], the enriched pathway includes 17 proteins whose functions in Treg cells have not yet been established. Interestingly, a previous proteomics study of human Treg and conventional T cells has shown an increased expression of glycolysis-related enzyme enolase 1 (ENO1) in Treg cells [18]. In our murine T cells, we found that although the expression level of ENO1 was higher than that of ENO3, ENO3 showed greater expression changes in iTreg cells, with a 2.3-fold increase compared to Th0 cells and a 2.6-fold increase when compared to Th17 cells. However, we did not observe significant expression changes of ENO1 in iTreg compared to Th0 cells, and only a minor increase (0.2-fold increase) was observed when comparing to Th17 cells. These results suggest that in murine Th17/iTreg cells, ENO3 but not ENO1 may have a more important role. Taken together, our data showed that a large number of proteins involved in metabolic pathways were differently expressed in Th17 and iTreg cells, suggesting that Th17 and iTreg cells use distinct energy resource to maintain their function.

The comparison of the proteomics and transcriptomics data from iTreg cells led to the recognition of the high differential expression of VIM in iTreg cells. VIM is the major cytoskeletal component of mesenchymal cells with important roles in cell adhesion, migration, differentiation, cytoskeletal rearrangements, and regulation of cell morphology. VIM has an important function in epithelial–mesenchymal transition and tumorigenesis [66–68]. In lymphocytes, VIM provides structural support in circulating human lymphocytes and also plays a role in lymphocyte adhesion and transcellular migration [69]. A recent study demonstrates that in a mouse model, VIM is required for TGFβ-induced wound healing [66]. In the present study, we found that VIM is highly expressed in iTreg cells, and TGFβ up-regulated the expression of VIM. Although Vim mRNA was also slightly up-regulated in Th17 cells, its protein expression level is significantly lower in Th17 cells compared to iTreg cells, suggesting that addition of the inflammatory cytokine interleukin 6 (IL6) in the cell culture may suppress TGFβ-induced VIM expression. Importantly, we found that VIM was involved in TGFβ-induced Foxp3 expression. However, this effect was prevented by IL2. Since both TGFβ and IL2 have fundamental functions in T cells, it would be interesting to further explore how the expression of VIM in T cells contributes to different immune-associated diseases. In the present study, we show that the metabolic pathway is one of the most enriched pathways in iTreg cells and that VIM is highly expressed in iTreg cells. Interestingly, a previous study has reported that VIM interacts with cytosolic phospholipase A2 α (cPLA2α) and functions as an adaptor of cPLA2α to function properly during the eicosanoid biosynthetic process [70]. It will also be important to further characterize the mechanisms by which VIM influences T cell functions and what signaling pathways are involved, especially whether it is involved in regulating T cell metabolism.

In summary, these proteomics data provide additional information to the transcriptomics data to help better characterization of Th17 cells, an important group of Th cells that are central to autoimmunity and inflammation. In addition, our dataset provides a valuable resource for further functional characterization of novel players involved in Th17/iTreg cell differentiation or function that could also lead to the development of novel therapeutic targets to modulate Th17/iTreg cells to treat human diseases.

## Materials and methods

### Ethics statement

Mice used in this study were maintained in the Central Animal Laboratory at Turku University. All experiments were carried out in accordance with appropriate guidelines for the care and use of laboratory animals and were approved by the Finnish Animal Ethics Committee.

### CD4+ T cell isolation and culture

Mouse splenocytes were isolated from 8–10-wk-old C57BL/6 mice. For VIM study, mouse splenocytes from 6–10-wk-old Vim−/−, and WT mice were isolated. Mice were maintained in the Central Animal Laboratory at Turku University. CD4+CD62L+ T cell Isolation Kit II (Miltenyi Biotec, Bergisch Gladbach, Germany) was used to obtain naïve T cells. The cells were activated with plate-bound anti-CD3 (1 μg/mL, BD PharMingen, San Diego, CA) and anti-CD28 (1 μg/mL) for all Th0, Th17, and iTreg conditions. For Th17 polarization, IL6 (30 ng/ml, PeproTech, UK), TGFβ1 (5 ng/ml), and neutralizing antibodies anti-IL4 and anti-IFNγ (both at 10 μg/mL, BD PharMingen, San Diego, CA) were added, and cells were cultured for 3 d. For iTreg conditions, IL2 (10 ng/ml, R&D system, Minneapolis, MN) and TGFβ1 (20 ng/ml) were used, and cells were cultured for 7 d, followed by restimulation with anti-CD3/CD28 and culturing with fresh cytokine-containing medium for another 3 d. All cell cultures were performed in IMDM supplemented with 10% fetal calf serum, 2 mM glutamine, 100 iu/mL penicillin, 0.1 mg/mL streptomycin (Sigma, St Louis, MO), and 2.5 μM β-mercaptoethanol.

### Sample preparation for mass spectrometric analysis and transcriptomics study

In vitro cultured murine CD4+ T cells were collected after 72 h or 10 d of polarization under polarizing conditions indicated above in “CD4+ T cell isolation and culture”. The cells were lysed with lysis buffer containing 0.5% (v/v) NP-40, 150 mM NaCl, and 50 mM Tris-HCl. The lysates were sonicated in Bioruptor Sonicator, and the supernatants were precipitated with Acetone. The obtained pellets were dissolved in 25 mM ammonium bicarbonate buffer containing 8 M Urea. The protein concentrations were measured using Bio-Rad DC protein assay kit. Proteins were reduced with DTT for 1 h at 37°C and alkylated with Iodoacetamide for 30 min in dark at room temperature and then were digested with trypsin (Promega sequencing grade). The trypsin to protein ratio of 1:30 (w/w) was used, and digestion was performed at 37°C overnight. The trypsin activity was quenched by adding 10% of triflouroacetic acid. Peptides were desalted with C18 tips (OMIX, Agilent) according to manufacturer’s instruction. The detergent was removed with HiPPR Detergent Removal Spin Column Kit (Thermo Scientific) according to manufacturer’s recommendations. The peptides were concentrated in Speedvac and resuspended in 2% formic acid and 2% acetonitrile before mass spectrometry analysis.

### LC-MS/MS analysis

The trypsin-digested peptides were analyzed by Q Exactive mass spectrometer (Thermo Fisher Scientific, Bremen, Germany) coupled to a nano-flow UHP-LC system (Easy-nLC1200, Thermo Fisher Scientific). Peptides (200 ng) were first loaded on a trapping column and subsequently separated on a C18 column (75 μm × 150 mm, 5 μm 200 Å, Dr. Maisch). The mobile phase consisted of a binary mixture of water and acetonitrile alone with formic acid.

The peptides were separated from 5% to 35% of solvent B in 85 min at a flow rate of 300 nl/min. The tandem mass spectra were acquired automatically by using Thermo Xcalibur software (Thermo Fisher Scientific). An information-dependent acquisition method with higher-energy C-trap dissociation (HCD) fragmentation of top 10 most intense ion in survey scan of mass range 300–1800 m/z was used. Samples were run in triplicate in randomized batches. To establish the consistent performance of the instrument, both a pooled sample and in-house standard digest were analyzed at the beginning and end of the batch.

### MS data analysis

The MS data raw files were searched against a mouse (*M*. *musculus*) UniProt database (downloaded on 18 January 2016) using the Andromeda search engine integrated into MaxQuant software version 1.5.3.30. The specified search parameters included carbamidomethyl (C) as a fixed modification and methionine oxidation and N-terminal acetylation as variable modification together with 1 tryptic missed cleavage. The peptide and protein FDR were set to 1%. Match between run options was enabled to transfer the identification across the samples.

### Proteomic data analysis

Samples for each condition (Thp, Th0, Th17, and iTreg) were derived from 3 cultures, all of which were analyzed using 3 technical replicates. Experimental design was paired. The nondetected MaxQuant intensities were imputed with the minimum nonzero intensity value of the corresponding technical replicate. Proteins MaxQuant identified only by site or labeled as potential contaminants were filtered out of the analysis. We preprocessed the data by summarizing technical replicates with medians, transformed the values to a logarithmic scale, and did the quantile normalization. We used Bioconductor package limma [71] to perform moderated *t* test with paired design to detect the differentially abundant proteins between iTreg and Th0 cells and Th17 and Th0 cells. Moderated *t* test with unpaired design was used to detect the differentially abundant proteins between iTreg and Thp cells as well as iTreg and Th17 cells. Statistical tests were done for such proteins that had at least 1 detected protein intensity value in both conditions. We performed the tests separately for the proteins detected in all samples and for the proteins that were not detected in some of the samples. For the latter proteins, we ignored samples whose corresponding sample pairs were not detected. By using Benjamini-Hochberg multiple correction, the differentially abundant proteins were identified with FDR < 0.05. Those proteins that had detected intensities in only 1 of the conditions were identified without statistical testing and were called as “selectively expressed.” All proteins that had at least 2 paired biological replicates are considered detected.

### Transcriptomic data analysis

After 72 h of polarization under polarizing conditions indicated above in “CD4+ T cell isolation and culture”, 4 independent in vitro cultured murine CD4+ cells were collected. RNAs from these samples were extracted (RNeasy kit, Qiagen). RNA-seq with pair-end 100-nt read length was performed at the Turku Center for Biotechnology with HiSeq 2000 instrument using Illumina TruSeq chemistry according to the manufacture’s instruction.

The RNA-seq data were mapped using Tophat (version 2.0.14) with default parameters to the mm10 mouse reference genome. Read counts were computed with HTSeq-count [72], with options stranded false, feature type exon, and sorted by name and mode union. Negative binomial generalized log-linear model to the read counts for each gene was fitted, and gene-wise statistical tests using pair-wise experimental design were performed with Bioconductor edgeR package [73]. The data were normalized using model-based scaling [74]. The tag-wise dispersions for the generalized linear models were empirical Bayes estimates with expression levels specified by a log-linear model [75]. Genes that did not have more than 1 count per million (cpm) at least in 4 samples were filtered out of the analysis.

### Pathway enrichment analysis

Hypergeometric tests and GSEAs of GO and KEGG pathways were performed using R package clusterProfiler [76]. In hypergeometric tests, all proteins with FDR < 0.05 were considered differentially regulated, and the background consisted of all proteins detected in samples involved in the corresponding comparison. For GSEA, we ranked the proteins by the common logarithm of adjusted *p*-value, and the sign was derived from the logarithmic fold change. Uniprot identifiers were mapped to Entrez identifiers using R package biomaRt [77, 78].

### Western blotting

Cells were lysed by adding Triton X-100 lysis buffer (50 mM Tris-HCl, pH 7.5, 150 mM NaCl, 0.5% Triton X-100, 5% Glycerol, 1% SDS)–containing protease and phosphatase inhibitors (Roche). The protein quantification was carried out by detergent-compatible protein assay kit (Bio-Rad, Hercules, CA), and 30 μg of protein was resolved in 12% SDS-PAGE gel. The antibodies used in this study are NFATC2, PSMB5, VIM (all from Cell Signaling), ENO3 (Sigma), FOXO1 (Immunoway), SMYD3 (Abcam), FOXP3 (eBioscience), and β-actin (CalBioChem).

### Flow cytometry

Cultured T cells were stained for surface expression of CD69 and CD101 (both from BD Biosciences, San Diego, CA). Detection of FOXP3+ or IL17-producing cells was determined by intracellular staining. Briefly, cells were stimulated for 4 h with PMA and ionomycin; after 2 h, GolgiStop (BD Biosciences) was added. Stimulated cells were fixed and permeabilized with Transcription Factor Staining Buffer Set (eBioscience, San Diego, CA) stained with anti-IL17-phycoerythrin (BD Biosciences), Foxp3-APC, or FOXP3-FITC (eBioscience) according to manufacturer’s instructions and detected in flow cytometer LSRII (Becton Dickinson, San Jose, CA). Events were collected and analyzed by using flowjo software (Tree Star, Ashland, OR).

**Key Learning Points**

- **The first proteomics characterization of Th17 cells**
- **Identification of proteins differentially expressed in mouse Th17 and iTreg cells**
- **Comparison of proteomic and transcriptomics changes in mouse Th17 and iTreg cells**

## Supporting information

S1 Fig Correlation of 3 biological replicates. (a) PCA plot of proteomes of different in vitro cultured subsets. Symbols represent biological replicates, and color code represents respective subsets. Ellipses were drawn manually. (b) Pearson’s correlation plots showing the correlation coefficient of 3 biological replicates for Thp. (c) Distribution of signal intensities. Plots show the expression of all quantified proteins over 6 orders of magnitude form different cell types. The complete lists of detected proteins can be found in S1 Data. PCA, principal component analysis; Thp, naïve CD4+ T cells.
(TIF)

S2 Fig Functional groups of DE proteins in Th17 versus Th0 or iTreg versus Th0 cells. (a) Heatmaps showing log-fold-change values with selected functional groups of DE proteins in comparison of Th17 versus Th0 and iTreg versus Th0 cells. Protein annotation for functional groups is obtained from IPA. The complete lists of DE proteins can be found in S2 Data. (b) KEGG pathway enrichment analysis was performed on DE proteins of Th17 versus Th0 conditions. The significantly (Fisher exact test, Benjamini-Hochberg adjusted *p* < 0.05) enriched pathways are presented in the plot, color indicates the adjusted *p*-value, and size of dot indicates the number of proteins enriched for that pathway. The list of pathways and proteins can be found in S2 Data. DE, differentially expressed; IPA, Ingenuity Pathway Analysis; iTreg, induced regulatory T cells; KEGG, Kyoto Encyclopedia of Genes and Genomes; Th0, T cell receptor–activated helper T cell; Th17, T helper 17
(TIF)

S3 Fig Distinct protein expression changes in Th17 and iTreg cells. (a) Venn diagram showing the comparisons of DE proteins between Th17 (Th17 versus Th0) and iTreg (iTreg versus Th0) cells. (b and c) Log-fold-change heat map of selected proteins of Th17 versus iTreg in comparison with Th0. The white color in the heat map indicates that protein differences were not statistically significant. The lists of DE proteins in Th17 versus iTreg cells are in S3 Data. (d) String network of protein interactions in Th17 and iTreg cells. The modules shown were obtained by performing the gene-set-enrichment analyses of GO and KEGG pathway. The colors of nodes indicate the log fold change of Th17 and iTreg proteins comparison, and size of nodes indicates the degree of connectivity of the nodes. The list of pathways and proteins can be found in S3 Data. DE, differentially expressed; GO, Gene Ontology; iTreg, induced regulatory T; KEGG, Kyoto Encyclopedia of Genes and Genomes; Th0, T cell receptor–activated helper T cell; Th17, T helper 17
(TIF)

S4 Fig Correlation of protein and RNA expression changes during Th17 differentiation. (a) Functional groups of DE proteins with and without consistent mRNA changes and DE mRNA without correlated protein changes (in S4 Data). (b) Genes that are regulated likewise in both transcriptomics and proteomics data have similar gene expression values as genes that are DE in transcriptomics data but not in proteomics data. The curves represent Gaussian kernel density estimates of base-2 logarithms of the mean FPKM values of Th17 samples (in S4 Data). DE, differentially expressed; FPKM, fragments per kilobase of transcript per million mapped reads; Th17, T helper 17
(TIF)

S5 Fig Immunoblot analysis of 2 replicates showing the validated proteins in Th0, iTreg, and Th17 cells. Immunoblot analysis showing the biological replicates for the expression of PSMB5, SMYD3, NFATC2, JUNB, ENO3, FOXO1, VIM, and loading control β-Actin. ENO3, enolase 3; FOXO1, forkhead box O1; iTreg, induced regulatory T cells; NFATC2, nuclear factor of activated T cells 2; PSMB5, proteasome subunit beta 5; SMYD3, SET and MYND domain containing 3; Th0, T cell receptor–activated helper T cell; Th17, T helper 17; VIM, vimentin.
(TIF)

S1 Data Lists of detected proteins and cumulative protein abundances in Th0, iTreg, Th17, and Thp cells, related to Fig 1, Fig 2A and S1 Fig. iTreg, induced regulatory T; Th0, T cell receptor–activated helper T cell; Th17, T helper 17; Thp, naïve CD4+ T cells.
(XLSX)

S2 Data Lists of DE proteins in Th17 versus Th0 and iTreg versus Th0 and pathways from pathway enrichment analysis, related to Fig 2 and S2 Fig. DE, differentially expressed; iTreg, induced regulatory T; Th0, T cell receptor–activated helper T; Th17, T helper 17
(XLSX)

S3 Data Lists of DE proteins in Th17 versus iTreg, pathways from pathway enrichment analysis and proteins for network analysis, related to Fig 3 and S3 Fig. DE, differentially expressed; iTreg, induced regulatory T; Th17, T helper 17.
(XLSX)

S4 Data Lists of DE genes in Th17 versus Th0 and iTreg versus Th0 cells with detected proteins, DE proteins with encoded genes, and correlated protein and mRNA expression changes, related to Fig 4A and 4B. DE, differentially expressed; iTreg, induced regulatory T; Th0, T cell receptor–activated helper T; Th17, T helper 17
(XLSX)

S5 Data The log fold change values of selected proteins and mRNA DE in Th17 and iTreg cells in comparison with Th0 cells and Th17 compared with iTreg cells, related to Fig 5A. DE, differentially expressed; iTreg, induced regulatory T; Th0, T cell receptor–activated helper T; Th17, T helper 17
(XLSX)

S6 Data mRNA expression of Vim in Th17, iTreg and Th0 cells and quantification of Foxp3 expression in Vim+/+ and Vim−/− mice, related to Fig 6A and 6D. Foxp3, forkhead box P3; iTreg, induced regulatory T; Th0, T cell receptor–activated helper T; Th17, T helper 17; Vim, vimentin.
(XLSX)

## Abbreviations

ACAA2: acetyl-CoA acyltransferase 2
Actg1: γ-actin 1
ADSSL1: adenylosuccinate synthetase isozyme 1
Aebp2: adipocyte enhancer–binding protein 2
AHR: aryl hydrocarbon receptor
ALDH2: aldehyde dehydrogenase 2
Aldoa: aldolase A
Arid1a: AT-rich interaction domain 1A
ATP5A1: ATP synthase F1 subunit alpha
ATP5B: ATP synthase subunit beta
Bach: BTB domain and CNC homology
BASP1: brain abundant membrane attached signal protein 1
Bcl10: B cell chronic lymphocytic leukemia/lymphoma 10
Brd8: bromodomain-containing 8
C: carbamidomethyl
C1qbp: complement C1q binding protein
Carm1: coactivator-associated arginine methyltransferase 1
Cbfb: core binding factor beta
Ccar1: cell cycle and apoptosis regulator 1
Ccnh: cyclin H
Ccnt1: cyclin T1
CCR7: C-C motif chemokine receptor 7
Cdc5l: cell division cycle 5–like protein
Chd4: chromodomain helicase DNA-binding protein 4
CLP1: cleavage and polyadenylation factor I subunit 1
CNS-1: conserved noncoding DNA sequence 1
Coa6: cytochrome c oxidase assembly factor 6
Cops2: constitutive photomorphogenesis 9 signalosome subunit 2
Coro1a: coronin 1A
COX2: cyclooxygenase 2
COX5A: cyclooxygenase 5A
CNOT2: CCR4-NOT transcription complex subunit 2
cPLA2α: cytosolic phospholipase A2 alpha
cpm: count per million
CPT1A: carnitine palmitoyltransferase 1A
CPT2: carnitine palmitoyltransferase 2
Ctla4: cytotoxic T lymphocyte–associated protein 4
Cxxc1: CXXC finger protein 1
Ddx5: DEAD-box helicase 5
DE: differentially expressed
Dnmt3a: DNA methyltransferase 3 alpha
Eif4a1: eukaryotic translation initiation factor 4a1
ENO1: enolase 1
ENO3: enolase 3
ETV6: ETS variant 6
FAM129B: family with sequence similarity 129 member B
Fasn: fatty acid synthase
FDR: false discovery rate
Foxk1: forkhead box K1
FOXO1: forkhead box O1
Foxp1: forkhead box P1
Foxp3: forkhead box P3
FPKM: fragments per kilobase of transcript per million mapped reads
FSCN1: fascin actin-bundling protein 1
Fus: Fus RNA binding protein
Gabpa: GA-binding protein transcription factor subunit alpha
Gapdh: glyceraldehyde-3-phosphate dehydrogenase
Gatad2a: GATA zinc finger domain containing 2A
Gimap4: GTPase IMAP family member 4
Gimap5: GTPase IMAP family member 5
GLUT1: glucose transporter 1
GLUT3: glucose transporter 3
GNG2: G protein unit gamma 2
GSEA: Gene Set Enrichment Analysis
Gtf2a1: general transcription factor IIA subunit 1
Gtf2e1: general transcription factor IIE subunit 1
H2afx: histone 2A family member X
HACD3: 3-hydroxyacyl-CoA dehydratase 3
HADH: hydroxyacyl-CoA dehydrogenase
HCD: higher-energy C-trap dissociation
Hcfc1: host cell factor C1
HDAC1: histone deacetylase 1
HDAC3: histone deacetylase 3
Hexim1: hexamethylene bisacetamide inducible 1
HIF1α: hypoxia-inducible factor 1 subunit alpha
Hist1h1e: histone cluster 1 H1 family member E
Hk1: hexokinase 1
Hmgb1: high-mobility group box 1
Hprt: hypoxanthine phosphoribosyltransferase
Hnrnpd: heterogeneous nuclear ribonucleoprotein D
Hnrnpk: heterogeneous nuclear ribonucleoprotein K
Hspd1: heat shock protein family D1
IBD: inflammatory bowel disease
ICAM1: intercellular adhesion molecule 1
ICAM2: intercellular adhesion molecule 2
Iigp1: interferon-inducible GTPase 1
IKZF3: IKAROS family zinc finger 3
IKZF4: IKAROS family zinc finger 4
IL2: interleukin 2
IL2RA: interleukin 2 receptor alpha
IL2RG: interleukin 2 receptor gamma
IL4r: interleukin 4 receptor
IL6: interleukin 6
IL16: interleukin 16
IL17: interleukin 17
IL17A: interleukin 17A
IL17F: interleukin 17F
IPA: Ingenuity Pathway Analysis
Irf3: interferon regulatory factor 3
IRF4: interferon regulatory factor 4
Isg15: interferon-stimulated gene 15
ITGAE: integrin subunit alpha E
ITGAL: integrin alpha L
ITGB1: integrin beta 1
ITGB2: integrin beta 2
ITGB7: integrin beta 7
iTreg: induced regulatory T
Jak3: Janus kinase 3
KEGG: Kyoto Encyclopedia of Genes and Genomes
Khdrbs1: KH RNA binding domain containing, signal transduction–associated 1
Lag3: lymphocyte activating 3
Lck: lymphocyte cell-specific protein tyrosine kinase
LC-MS: mass spectrometry coupled with liquid chromatography
LC-MS/MS: liquid chromatography–tandem mass spectrometry
LFQ: label-free quantitative
Lgals3: galectin 3
Lgals7: galectin 7
MALT1: mucosa-associated lymphoid tissue 1
MAPK11: mitogen-activated protein kinase
Max: MYC-associated factor X
Mecp2: methyl-CpG binding protein 2
Mif: macrophage migration inhibitory faction
Mta2: metastasis-associated 1 family member 2
Mybbp1a: MYB binding protein 1A
Myh9: myosin heavy chain 9
MYH11: myosin heavy chain 11
NAFLD: nonalcoholic fatty liver disease
Ncl: nucleolin
NCoR: nuclear receptor corepressor
NDUFS1: NADH:ubiquinone oxidoreductase core subunit S1
NFAT1, TF NFATC2, NFATC2: nuclear factor of activated T cells 2
NFKB1: nuclear factor kappa B subunit 1
Nfkbia: nuclear factor of kappa light polypeptide gene enhancer in B cells inhibitor alpha
Nfyb: nuclear transcription factor Y beta
Nmi: N-myc and STAT interactor
NR3C1: nuclear receptor subfamily 3 group C member 1
nTreg: natural regulatory T
OXPHO: oxidative phosphorylation
P4HA1: prolyl 4-hydroxylase subunit alpha 1
PDE5A: phosphodiesterase 5A
Phb: prohibitin
Phb1: prohibitin 1
Phb2: prohibitin 2
Phf5a: PHD finger protein 5a
Pkm: pyruvate kinase M
Pml: promyelocytic leukemia
Ppia: peptidylprolyl isomerase A
Pqpb1: polyglutamine-binding protein 1
PXN: paxillin
Psmd9: proteasome 26S subunit, non-ATPase 9
Rac2: ras-related C3 botulinum toxin substrate 2
Ran: Ras-related nuclear protein
Rbbp7: retinoblastoma binding protein 7
Rbm39: RNA binding motif 39
REXO4: RNA exonuclease 4
RIOK1: right open reading frame kinase 1
ROMO1: reactive oxygen species modulator 1
RORγt: retinoic acid receptor–related orphan receptor gamma T
Rorc: retinoic acid receptor–related orphan receptor C
RUNX1: runt-related transcription factor 1
RUNX3: runt-related transcription factor 3
Ruvbl1: RuvB-like AAA ATPase 1
Ruvbl2: RuvB-like AAA ATPase 2
Sap130: Sin3A-associated protein 130
SATB1: special AT-rich sequence-binding protein 1
Serpinb5: Serpin Family B Member 5
SIRT2: sirtuin 2
SLC2A1: solute carrier family 2 member 1
SLC2A3: solute carrier family 2 member 3
SLC4A2: solute carrier family 4 member 2
Slc25a2: solute carrier family 25 member 2
SMARCA4: SWI/SNF-related matrix-associated actin-dependent regulator of chromatin subfamily A member 4
SMARCA5: SWI/SNF-related matrix-associated actin-dependent regulator of chromatin subfamily A member 5
SMARCB1: SWI/SNF-related matrix-associated actin-dependent regulator of chromatin subfamily B member 1
SMARCC2: SWI/SNF-related matrix-associated actin-dependent regulator of chromatin subfamily C member 2
Smarcd: SWI/SNF-related matrix-associated actin-dependent regulator of chromatin subfamily D
SMARCD1: SWI/SNF-related matrix-associated actin-dependent regulator of chromatin subfamily D member 1
SMARCE1: SWI/SNF-related matrix-associated actin-dependent regulator of chromatin subfamily E member 1
SMRT: silencing mediator for retinoid and thyroid receptors
SMYD3: SET and MYND domain containing 3
Sqstm1: sequestosome 1
Srsf2: serine- and arginine-rich splicing factor 2
Stat1: signal transducer and activator of transcription 1
Stat2: signal transducer and activator of transcription 2
STAT3: signal transducer and activator of transcription 3
Stat5a: signal transducer and activator of transcription 5A
Stat6: signal transducer and activator of transcription 6
Stip1: stress-induced phosphoprotein 1
STMN2: stathmin 2
Supt16h: SPT16 homolog
Tbl1xr1: transducin beta like 1 X-linked receptor 1
Tcea1: transcription elongation factor A polypeptide 1
Tceb3: transcription elongation factor B polypeptide 3
Tcf7: transcription factor 7
TCR: T cell receptor
TF: transcription factor
TGFβ: transforming growth factor beta
TGFβR: transforming growth factor beta receptor type 1
TGM2: transglutaminase 2
Th0: T cell receptor–activated helper T
Th1: T helper 1
Th2: T helper 2
Th17: T helper 17
Thoc1: THO complex 1
Thp: naïve CD4+ T
TNFRSF4: tumor necrosis factor receptor superfamily member 4
TNFRSF18: tumor necrosis factor receptor superfamily member 18
Traf6: tumor necrosis factor receptor–associated factor 6
Treg: regulatory T
Trrap: transformation/transcription domain–associated protein
Uba1: ubiquitin 1
Uba52: ubiquitin 52
Ube2v1: ubiquitin conjugating enzyme E2 V1
Ubtf: upstream binding transcription factor
VIM: vimentin
Wdr1: WD repeat domain 1
WT: wild-type
Yy1: yin yang 1
Zap70: zeta chain of T cell receptor–associated protein kinase 70
