# Supplementary figures and images for: Quantitative proteomic characterization and comparison of T helper 17 and induced regulatory T cells
Source: PLoS Biol. 2018 May 31;16(5):e2004194. doi: 10.1371/journal.pbio.2004194 (PMC5979006; doi:10.1371/journal.pbio.2004194)

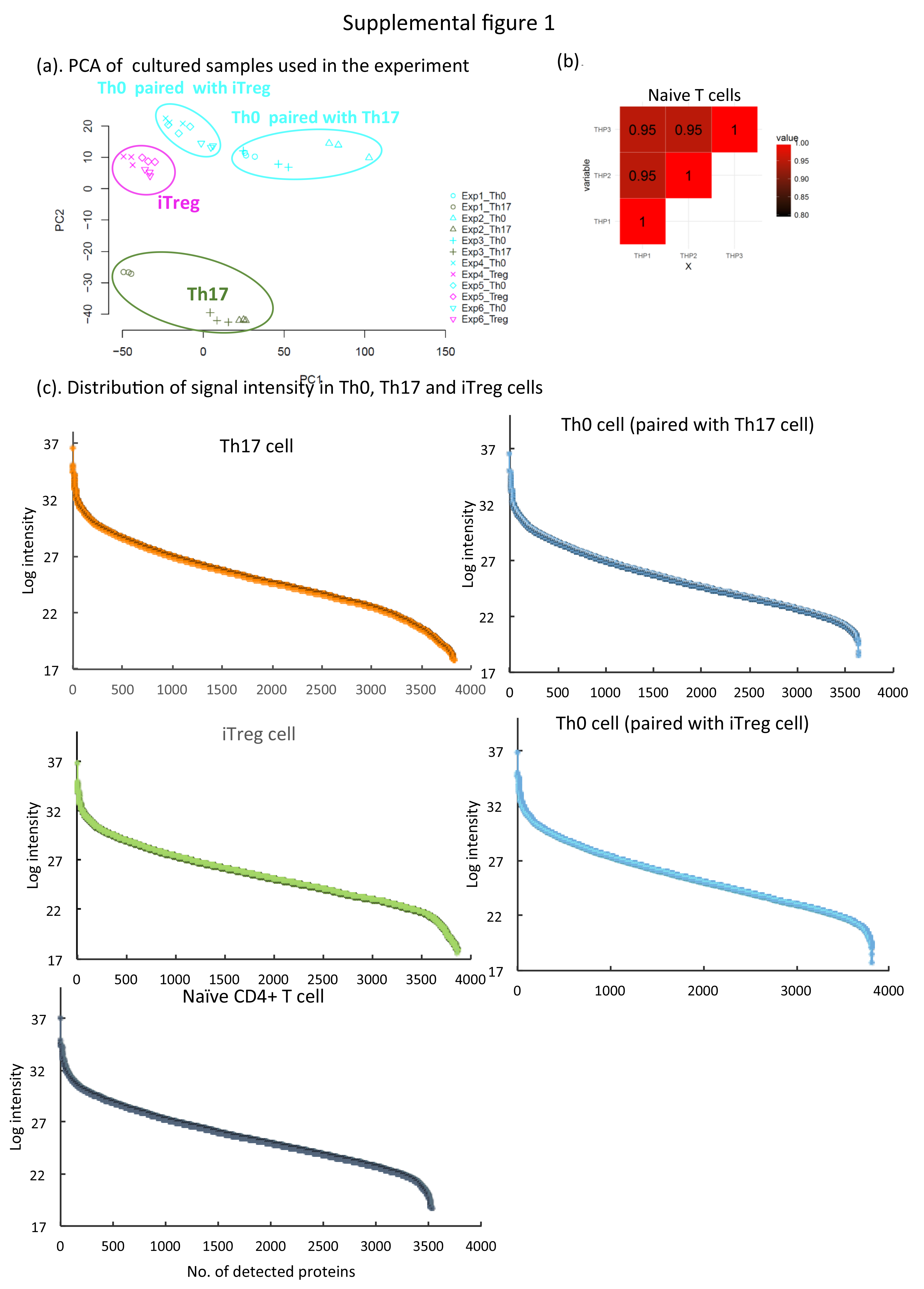

Supplement: S1 Fig — (a) PCA plot of proteomes of different in vitro cultured subsets. Symbols represent biological replicates, and color code represents respective subsets. Ellipses were drawn manually. (b) Pearson’s correlation plots showing the correlation coefficient of 3 biological replicates for Thp. (c) Distribution of signal intensities. Plots show the expression of all quantified proteins over 6 orders of magnitude form different cell types. The complete lists of detected proteins can be found in S1 Data. PCA, principal component analysis; Thp, naïve CD4+ T cells. (TIF) [file pbio.2004194.s001.tif]

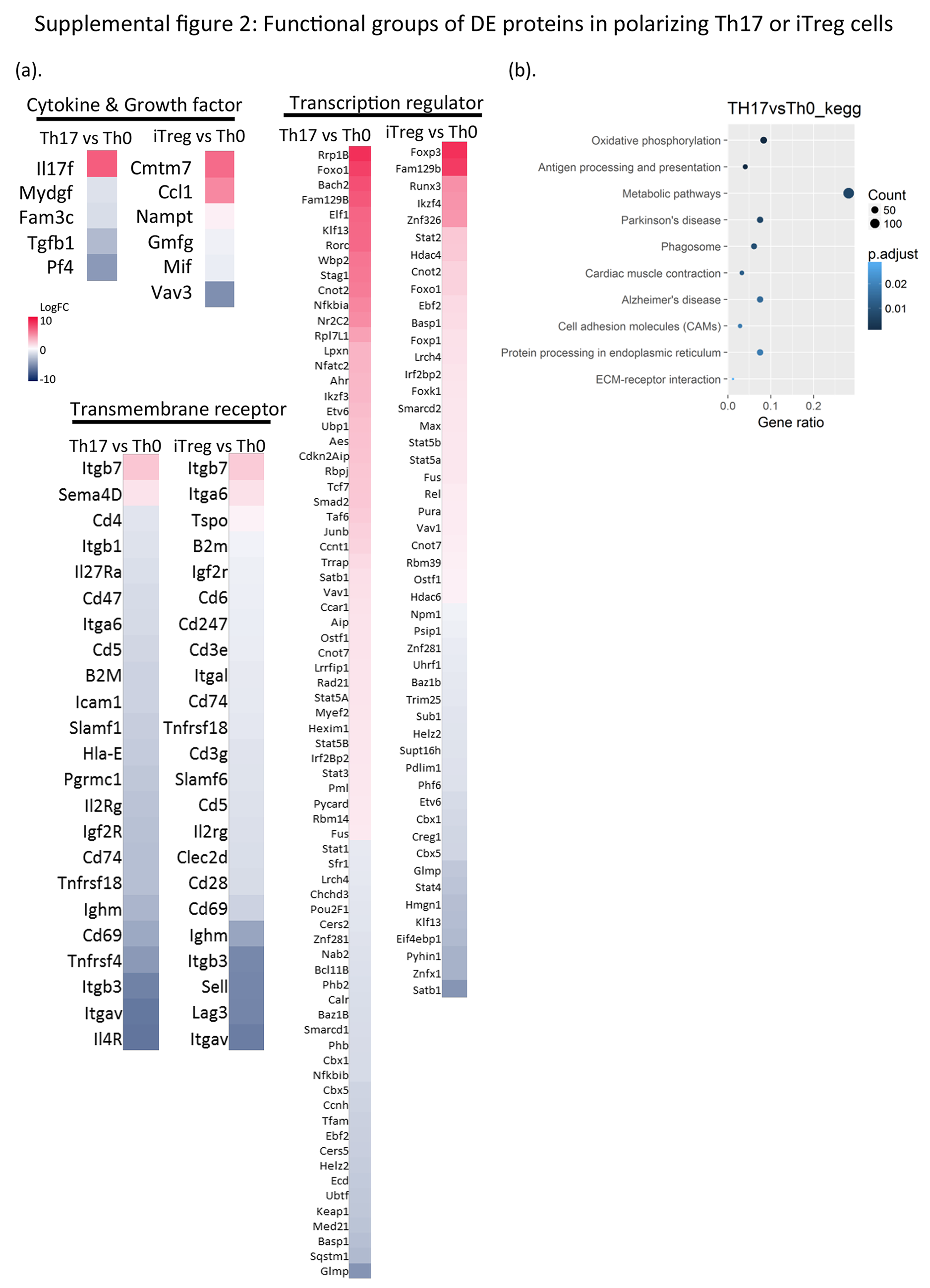

Supplement: S2 Fig — (a) Heatmaps showing log-fold-change values with selected functional groups of DE proteins in comparison of Th17 versus Th0 and iTreg versus Th0 cells. Protein annotation for functional groups is obtained from IPA. The complete lists of DE proteins can be found in S2 Data. (b) KEGG pathway enrichment analysis was performed on DE proteins of Th17 versus Th0 conditions. The significantly (Fisher exact test, Benjamini-Hochberg adjusted p < 0.05) enriched pathways are presented in the plot, color indicates the adjusted p-value, and size of dot indicates the number of proteins enriched for that pathway. The list of pathways and proteins can be found in S2 Data. DE, differentially expressed; IPA, Ingenuity Pathway Analysis; iTreg, induced regulatory T cells; KEGG, Kyoto Encyclopedia of Genes and Genomes; Th0, T cell receptor–activated helper T cell; Th17, T helper 17 (TIF) [file pbio.2004194.s002.tif]

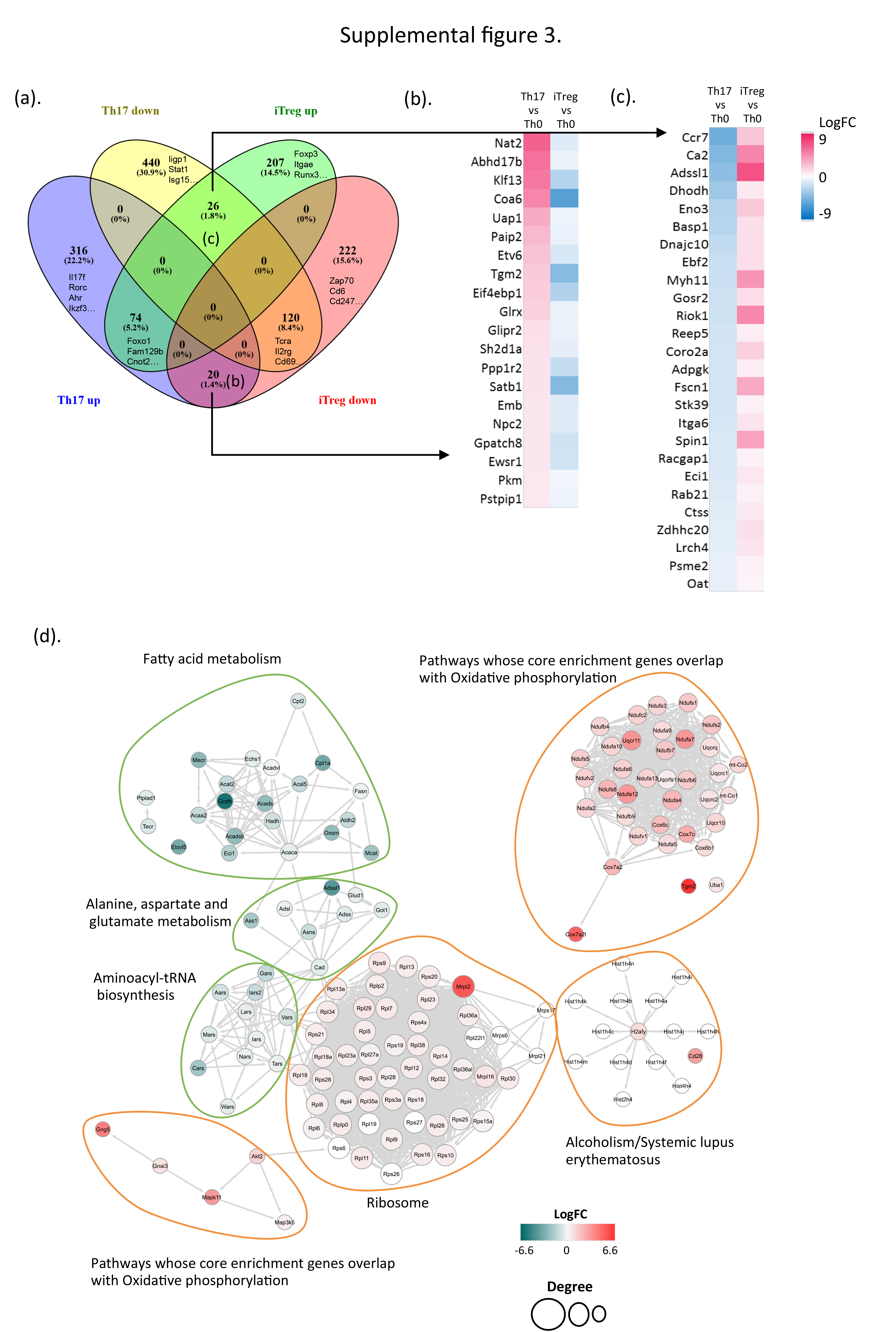

Supplement: S3 Fig — (a) Venn diagram showing the comparisons of DE proteins between Th17 (Th17 versus Th0) and iTreg (iTreg versus Th0) cells. (b and c) Log-fold-change heat map of selected proteins of Th17 versus iTreg in comparison with Th0. The white color in the heat map indicates that protein differences were not statistically significant. The lists of DE proteins in Th17 versus iTreg cells are in S3 Data. (d) String network of protein interactions in Th17 and iTreg cells. The modules shown were obtained by performing the gene-set-enrichment analyses of GO and KEGG pathway. The colors of nodes indicate the log fold change of Th17 and iTreg proteins comparison, and size of nodes indicates the degree of connectivity of the nodes. The list of pathways and proteins can be found in S3 Data. DE, differentially expressed; GO, Gene Ontology; iTreg, induced regulatory T; KEGG, Kyoto Encyclopedia of Genes and Genomes; Th0, T cell receptor–activated helper T cell; Th17, T helper 17 (TIF) [file pbio.2004194.s003.tif]

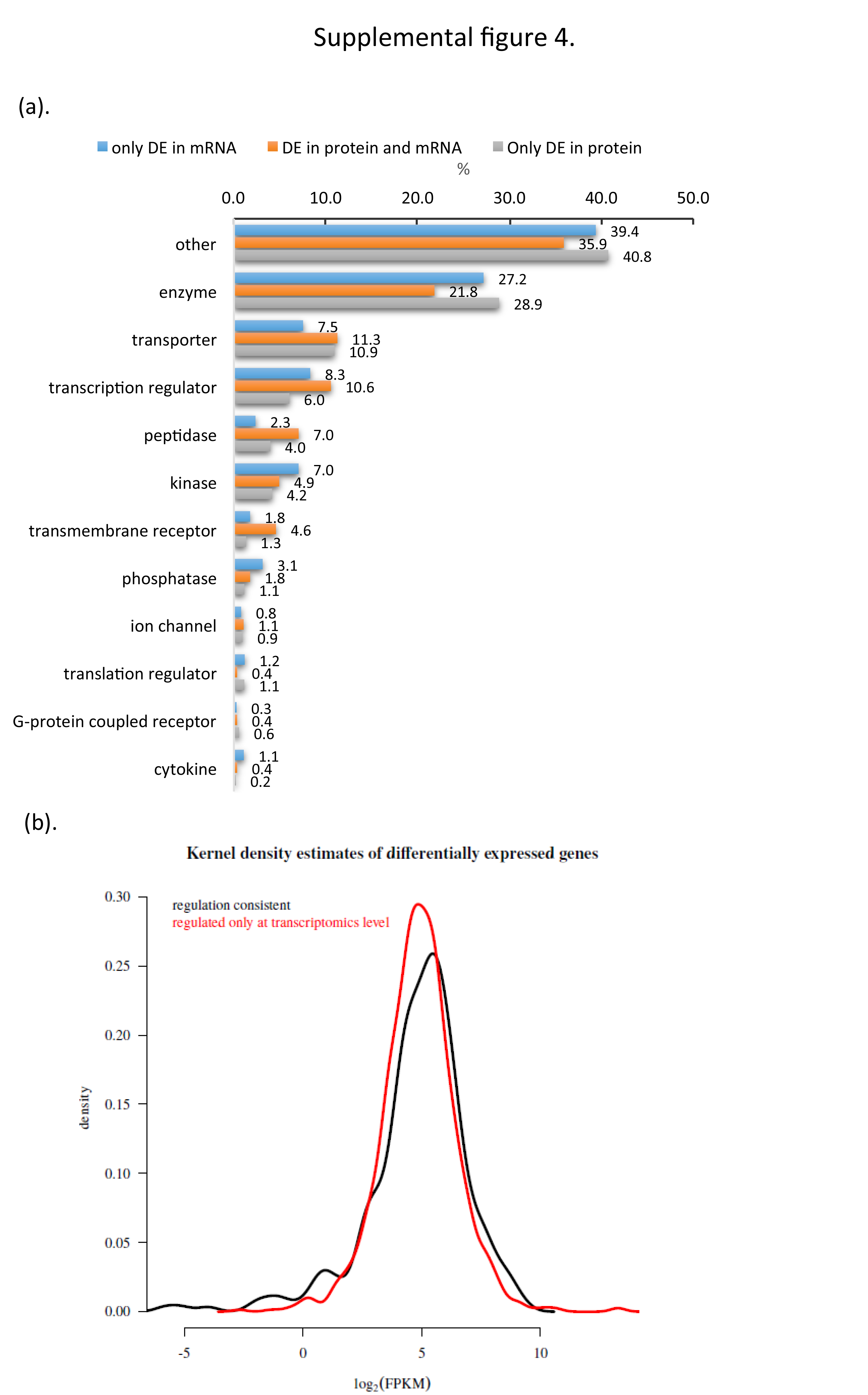

Supplement: S4 Fig — (a) Functional groups of DE proteins with and without consistent mRNA changes and DE mRNA without correlated protein changes (in S4 Data). (b) Genes that are regulated likewise in both transcriptomics and proteomics data have similar gene expression values as genes that are DE in transcriptomics data but not in proteomics data. The curves represent Gaussian kernel density estimates of base-2 logarithms of the mean FPKM values of Th17 samples (in S4 Data). DE, differentially expressed; FPKM, fragments per kilobase of transcript per million mapped reads; Th17, T helper 17 (TIF) [file pbio.2004194.s004.tif]

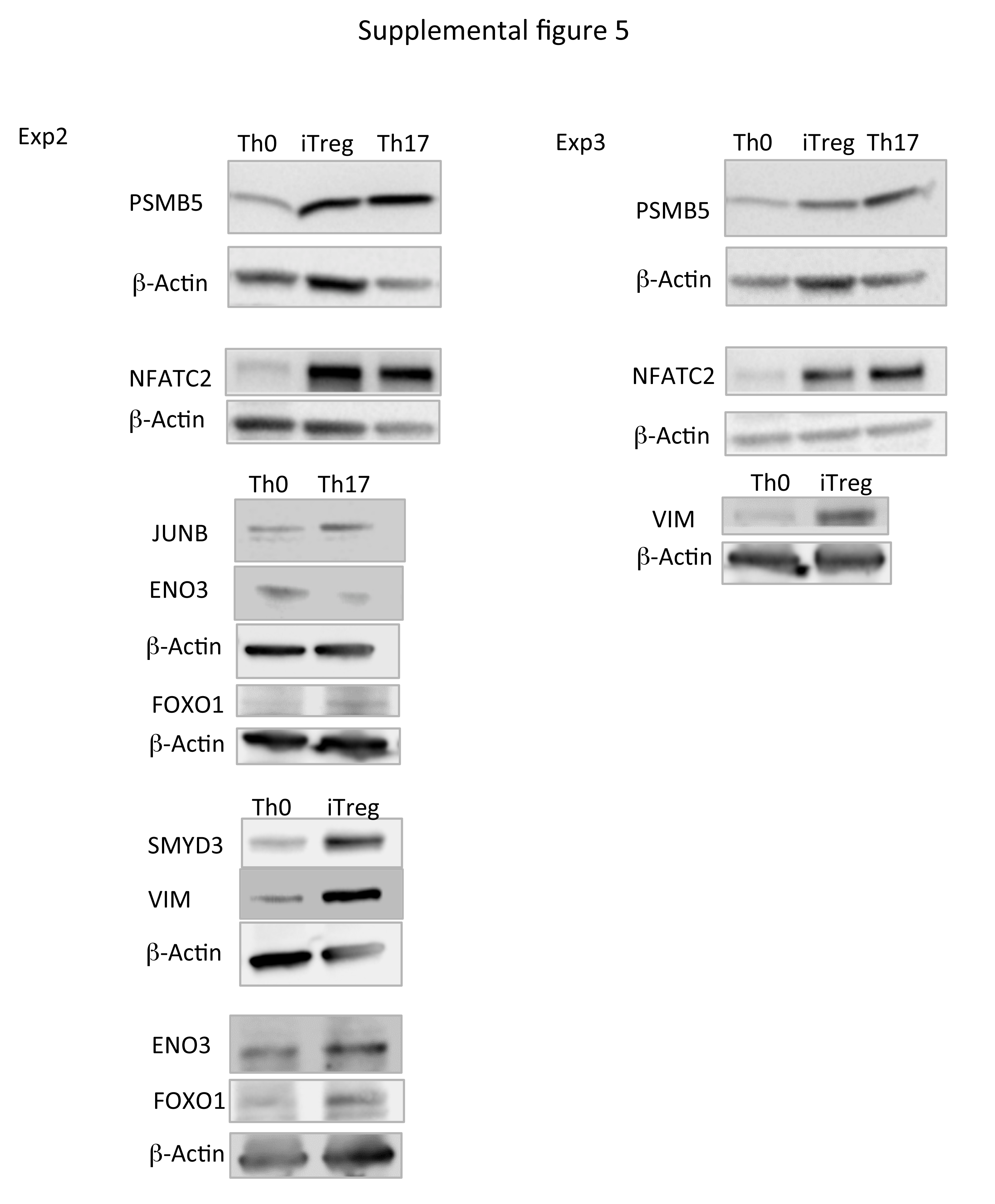

Supplement: S5 Fig — Immunoblot analysis showing the biological replicates for the expression of PSMB5, SMYD3, NFATC2, JUNB, ENO3, FOXO1, VIM, and loading control β-Actin. ENO3, enolase 3; FOXO1, forkhead box O1; iTreg, induced regulatory T cells; NFATC2, nuclear factor of activated T cells 2; PSMB5, proteasome subunit beta 5; SMYD3, SET and MYND domain containing 3; Th0, T cell receptor–activated helper T cell; Th17, T helper 17; VIM, vimentin. (TIF) [file pbio.2004194.s005.tif]
